# Diagnostic trajectories and stability of mental disorders in childhood and adolescence – A nation-wide cohort study using sequence analysis

**DOI:** 10.1192/j.eurpsy.2025.10091

**Published:** 2025-08-26

**Authors:** Mette Falkenberg Krantz, Søren Dalsgaard, Merete Osler, Martin Balslev Jorgensen, Anders Jorgensen, Terese Sara Høj Jørgensen

**Affiliations:** 1 Child and Adolescent Mental Health Center, Copenhagen University Hospital – Mental Health Services CPH, Copenhagen, Denmark; 2 Department of Clinical Medicine, University of Copenhagen, Copenhagen, Denmark; 3 National Centre for Register-Based Research, Aarhus University, Aarhus, Denmark; 4 Center for Clinical Research and Prevention, Bispebjerg and Frederiksberg Hospitals, Frederiksberg, Denmark; 5 Section of Epidemiology, Department of Public Health, University of Copenhagen, Copenhagen, Denmark; 6 Psychiatric Center Copenhagen, Rigshospitalet, Copenhagen, Denmark; 7 Section of Social Medicine, Department of Public Health, University of Copenhagen, Copenhagen, Denmark

**Keywords:** adolescent, child, mental disorder, stability, trajectory

## Abstract

**Background:**

Little is known about the diagnostic trajectories following a first psychiatric diagnosis in childhood or adolescence. Such knowledge could aid clinicians in treatment, risk prediction, and psychoeducation. This study presents a comprehensive nationwide overview of diagnostic trajectories in children and adolescents after their first diagnosis in child and adolescent psychiatric hospitals.

**Methods:**

Patients aged 0 to 17 years who received their first psychiatric diagnosis between January 1996 and December 2011 were identified through the Danish National Patient Registries. Shifts at the International Classification of Diseases (ICD-10) two-cipher level (F00-F99), grouped into 19 categories, were identified. Subsequent diagnoses during 10 years of follow-up until December 2021 were identified and analyzed using state sequence analysis and Cox proportional hazard regression models.

**Results:**

A total of 77,464 children and adolescents (32,733 [42.26%] girls) were identified with a first-time psychiatric diagnosis. Among these, 46.7% of girls and 37.6% of boys had at least one diagnostic shift after 10 years of follow-up. High entropy and low diagnostic stability were found in first-time diagnoses often presenting in adolescence, such as affective disorders, psychotic illness, and personality disorders, while lower entropy and high diagnostic stability were found in neurodevelopmental disorders and eating disorders. For most categories, girls had higher mean entropy measures than boys (*P* < 0.05).

**Conclusions:**

Diagnostic shifts are common in child and adolescent psychiatric services, particularly when the first contact occurs in adolescence. Adequate focus on psychoeducation about emerging diagnostic shifts, and on timely detection, particularly in girls, and particularly in adolescence, is warranted.

## Introduction

The prevalence of diagnosed mental disorders among children and adolescents has increased substantially worldwide over the past decades [1, 2]. Today, up to 15% of children and adolescents fulfill the criteria for a mental disorder before they turn 18 years old [3–5]. Childhood is a vulnerable period for mental disorders with distinct patterns of psychopathology and worse prognostic outcomes compared with adulthood onset [6, 7]. Prior mental disorder in childhood is considered to be the strongest predictor of later new episodes with the same mental disorder and is likewise a strong predictor of later development of other mental disorders [8]. However, knowledge concerning trajectories in diagnostic transitions across mental disorders following a first diagnosis in childhood and adolescence is sparse. While a few studies have assessed diagnostic trajectories with a focus on one or few subsequent psychiatric disorders, e.g. autism spectrum disorder (ASD) or attention-deficit hyperactivity disorder (ADHD), fewer have assessed trajectories across the full spectrum of diagnostic categories used in child and adolescent psychiatric services, and sample sizes and sex-specific analyses have been limited [7–11]. Recently, substantial diagnostic instability across the psychiatric diagnostic spectrum in adults was uncovered [12]. Likewise, knowledge concerning diagnostic stability, transition, and trajectories following a first diagnosis in childhood and adolescence is essential to increase the understanding of developmental psychopathology. Such knowledge can inform clinicians in service planning, treatment, and health policy decisions in the evaluation of needs for prevention and treatment [7].

In this study, we aimed to map diagnostic trajectories from the first psychiatric diagnosis during childhood (1–17 years old) and 10 years onward in the entire Danish population over more than two decades. Through sequence analyses with entropy measurements and risk estimates of subsequent change into other diagnostic groups, we provide a comprehensive overview of the temporal-diagnostic landscape that emerges after the first presentation of a mental disorder during childhood or adolescence.

## Methods

### Participants and data sources

A cohort consisting of all individuals in Denmark with a first-time in- or outpatient psychiatric hospital contact between January 1996 and December 2011 were identified in the Danish National Patient Registry [13, 14]. This register contains information on all psychiatric hospital admissions in Denmark since 1995, including data on outpatient treatment and emergency room contacts. We included all individuals aged less than 18.0 years with a discharge diagnosis of a mental or behavioral disorder corresponding to the International Statistical Classification of Disease and Related Health Problems 10th revision (ICD-10) codes as the first-time main diagnosis (denoted D1) in the period 1996–2011, enabling up to 10 years of subsequent diagnosis (denoted SD) follow-up in the registers until December 31, 2021 [15]. Only main, and not concurrent secondary, diagnoses were included. We linked the National Patient Register with the Danish Civil Registration System and the Population Education Register [16] to identify information on birthday, sex, emigration, and death [17]. The study was approved by the Regional Data Protection Agency.

### Definitions of first-time main diagnosis (D1) and subsequent diagnoses (SD)

We identified D1s across the full diagnostic spectrum, defined at the two-cipher level (F00–F99), which we grouped into 19 categories, in accordance with previous studies (presented in Table 1) [4].

**Table 1.** The distribution of first-time psychiatric hospital discharge diagnoses (D1) for all admissions among children and adolescents in Denmark and its relation to subsequent alternative diagnoses (SD) and deaths, 1996–2011, with 10 years follow-up

D1 code	Name	Girls					Boys				
		*N* (%)	Mean age (sd)	*N* (%) 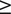 1 SD^a^	Mean SDs	*N* (%) Death	*N* (%)	Mean age (sd)	*N* (%) 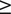 1 SD	Mean SDs	*N* (%) Death
F10-F19	Substance use disorder (SUD)	305 (0.9)	15.8 (1.3)	176 (57.7)	1.02 (1.14)	<= 5	589 (1.3)	16.2 (1.1)	317 (53.8)	0.94 (1.13)	17 (2.9)
F20–29	Schizophrenia spectrum disorder (SZ)	817 (2.5)	14.9 (2.0)	540 (66.1)	1.14 (1.10)	9 (1.1)	835 (1.9)	14.7 (2.7)	504 (60.4)	0.90 (0.94)	18 (2.2)
F30–31	Bipolar disorder (BD)	69 (0.2)	15.6 (1.7)	44 (63.8)	1.09 (1.15)	<= 5	63 (0.1)	15.6 (1.8)	43 (68.3)	1.11 (1.05)	<= 5
F32 + F33	Single and recurrent depression (SRD)	3056 (9.3)	15.2 (1.8)	1853 (60.6)	1.06 (1.14)	15 (0.5)	1358 (3.0)	14.5 (2.4)	804 (59.2)	0.96 (1.05)	16 (1.2)
F34-F39	Other mood disorders (OMD)	160 (0.5)	14.7 (2.2)	112 (70.0)	1.31 (1.26)	<= 5	108 (0.2)	13.7 (2.8)	62 (57.4)	0.92 (1.05)	<= 5
F40 + F41 + F93	Anxiety disorders (AND)	2253 (6.9)	12.2 (3.7)	1023 (45.4)	0.75 (1.04)	<= 5	2152 (4.8)	10.6 (3.6)	860 (40.0)	0.57 (0.84)	10 (0.5)
F42	Obsessive-compulsive disorder (OCD)	1341 (4.1)	12.5 (2.9)	530 (39.5)	0.61 (0.94)	<= 5	1197 (2.7)	11.9 (3.0)	443 (37.0)	11.9 (3.0)	<= 5
F43	Stress and adjustment disorders (SAD)	7543 (23.0)	13.7 (3.9)	3518 (46.6)	0.78 (1.05)	36 (0.5)	3749 (4.8)	11.6 (5.0)	1494 (39.9)	0.61 (0.90)	43 (1.2)
F44-F48	Somatoform disorders (SD)	294 (0.9)	13.7 (2.5)	145 (49.3)	0.90 (1.20)	<= 5	145 (0.3)	13.5 (3.0)	61 (42.1)	0.66 (0.97)	<=5
F50	Eating disorders (ED)	3900 (11.9)	14.6 (2.0)	1344 (34.5)	0.57 (0.96)	21 (0.5)	329 (0.7)	13.4 (2.7)	103 (31.3)	0.50 (0.92)	<= 5
F60–69	Personality disorders (PD)	1401 (4.3)	15.5 (1.5)	759 (54.2)	0.85 (0.99)	7 (0.5)	512 (1.1)	15.3 (2.2)	267 (52.2)	0.77 (0.94)	11 (2.2)
F70–79	Intellectual disability (ID)	897 (2.7)	10.0 (4.6)	334 (37.2)	0.52 (0.81)	14 (1.6)	1901 (4.2)	9.0 (4.2),	705 (37.1)	0.46 (0.68)	17 (0.9)
F80–83	Other developmental disorders (ODD)	848 (2.6)	9.5 (4.2)	354 (41.8)	9.5 (4.2)	<= 5	2769 (6.2)	8.5 (3.6)	1094 (39.5)	0.51 (0.73)	14 (0.5)
F84 (minus F84.2-F84.4)	Autism spectrum disorder (ASD)	1989 (6.1)	8.9 (4.5)	716 (36.0)	0.52 (0.84)	9 (0.5)	8625 (19.3)	8.1 (4.0)	2347 (27.2)	0.33 (0.61)	24 (0.3)
F90 (F90 + F98.8)	ADHD	3162 (9.7)	10.4 (3.9)	1190 (37.6)	0.58 (0.91)	<= 5	11031 (24.7)	9.5 (3.5)	2947 (26.7)	0.34 (0.64)	43 (0.4)
F91	Conduct disorders (CD)	460 (1.4)	11.6 (3.9)	268 (58.3)	1.01 (1.17)	<= 5	1634 (3.7)	10.4 (3.6)	850 (52.0)	0.76 (0.93)	19 (1.2)
F94 (minus F94.0)	Attachment disorders (AD)	941 (2.9)	8.7 (4.5)	417 (44.3)	0.73 (1.05)	<= 5	1642 (3.7)	8.4 (3.9)	684 (41.7)	0.56 (0.78)	<= 5
F95 (F95)	Tic disorders (TD)	158 (0.5)	9.9 (3.1)	80 (50.6)	0.78 (0.95)	<= 5	805 (1.8)	9.9 (2.9)	318 (39.5)	0.54 (0.80)	<= 5
F99 (F51-F59, F84.2-F84.4, F88, F89, F92, F98.0-F98.6, F98.9, F99.9)	Other	3139 (9.6)	10.7 (5.0)	1870 (59.6)	0.98 (1.08)	14 (0.5)	5287 (11.8)	9.1 (4.1)	2917 (55.2)	0.77 (0.85)	28 (0.5)
All		32733 (100.0)	12.6 (4.2)	15273 (46.7)	0.76 (1.03)	151 (0.5)	44731 (100.0)	9.9 (4.2)	16820 (37.6)	0.52 (0.79)	279 (0.6)

a A subsequent diagnosis could be any new diagnosis which differed from the first diagnosis. Thus, for someone with a first diagnosis of depression, any new diagnosis of depression would count as one if the diagnosis was not altered whereas a new diagnosis of bipolar disorder would count as a subsequent diagnosis.

The same categories were included as SDs, which also included the possibility of receiving the same diagnosis again, emigration, and death as subsequent events. For the purpose of the Cox proportional hazard analyses, SDs were extracted at the 1-cipher level (F0–F9, see Statistics) where the D1s were the exposure variables, and each SD was included as a separate outcome variable.

### Statistics

Sequence analysis is an evolving tool to describe longitudinal sets of individual categorical states in a population [18, 19]. For the purpose of the state sequence analysis, we assigned an SD to each state with a length of 12 months during 10 years of follow-up (equal to 10 12-month periods) following each D1. The SD was defined as the last diagnosis given within each 12-month period. We chose this strategy because the last diagnosis given within a period should have higher clinical validity than previous diagnoses. The prevailing diagnosis (D1 or SD) was projected to the following 12-month periods until an alternative SD was made. The use of 12-month periods was based on our judgment of a relevant clinical timespan for disease development and on preliminary analysis using 3- and 6-month periods, which were limited by inconsistent variations. Due to the European General Data Protection Regulation and rules of Statistics Denmark, sequences with less than five individuals were not presented in the graphs or text, but otherwise we attempted to depict and analyze the maximal complexity of sequence sets for each D1 by allowing the maximal number of sequences in each graph. To measure the diversity of the states (diagnostic categories) in a given sequence, we calculated the normalized entropy of each sequence, which can be interpreted as a measure of the diversity of states in a given sequence. The normalized entropy is the entropy divided by the entropy of the alphabet (i.e., all the states that can occur) and, thus, ranges from 0 to 1. The entropy of the alphabet is an upper bound of the entropy that corresponds to the maximal possible entropy when the sequence length is a multiple of the alphabet size. The normalized entropy has a maximal value of 1. If all states in the sequence are the same, the entropy is equal to zero [19]. We conducted the analyses for all girls and boys, respectively, and additionally performed t-tests and ANOVA tests to evaluate the difference in the mean entropy values based on age (0–10 and 11–18 years) and highest parental educational attainment (short (primary education and no official education in Denmark), medium (secondary and vocational education), and long (higher education).

Patients were followed from the date of their first contact until each of the SDs, respectively: death, emigration, or end of follow-up (10 years after study entry). In each analysis, the specific D1 was compared to all other D1s as the exposure variable. Supplementary Cox proportional hazard regression models were also performed. Due to multiple testing, we applied Bonferroni correction and used a significance level of 0.026% (5%/180 tests), corresponding to 99.9% confidence intervals.

Data preparation was made in SAS and analyses were performed in SAS 9.4 and R 4.1.3. with the “TraMineR” package installed [18, 19].

## Results

A total of 77,464 patients (32,733 girls, 44,731 boys) below 18 years of age diagnosed with a mental disorder after one or more contacts in an inpatient or outpatient psychiatric hospital setting from 1996 through 2011 were identified (Table 1).

### First time main diagnosis (D1)

For girls, the three most common D1s were stress and adjustment disorder (SAD) (23.0% of all D1s), eating disorders (ED) (11.9% of all D1s), and ADHD (9.7% of all D1s), and for boys, ADHD (24.7% of all D1s), ASD (19.3% of all D1s), and other developmental disorders (ODD) (6.2% of all D1s). Across the included 19 D1s, the diagnoses with the earliest age of onset were attachment disorder (AD) and ASD (mean age range from 8.1 to 8.9 years old) for both sexes. The diagnoses with the latest mean age onset were substance use disorder (SUD) and bipolar disorder (BD) (mean age range from 15.6 to 16.2 years old) for both sexes. The smallest D1 category among girls and boys was the BD category (*N* = 69 for girls and 63 for boys). For all D1s except for SUD (and bipolar and tic disorder where mean ages were equal between the sexes), boys were diagnosed at an earlier age than girls (Table 1).

### Subsequent diagnoses (SD)

Across all D1s, a total of 46.7% of girls and 37.6% of boys experienced at least one diagnostic shift (Table 1). Excluding the mixed “other” category, the three first-time diagnostic categories with the largest proportions of patients with at least one SD were, for girls, other mood disorders (OMD) (70.0%), schizophrenia spectrum disorder (SZ) (66.1%), and BD (63.8%), and for boys, BD (68.3%), schizophrenia spectrum disorder (60.4%), and single and recurrent depression (SRD) (59.2%). For girls, the first-time diagnostic category with the smallest proportion of patients with at least one SD was ED (34.5%), and for boys, the first-time diagnostic category with the smallest proportion of SDs was ADHD (26.7%) (Table 1).

### Entropy across psychiatric diagnoses in childhood and adolescence

**Figure 1.**
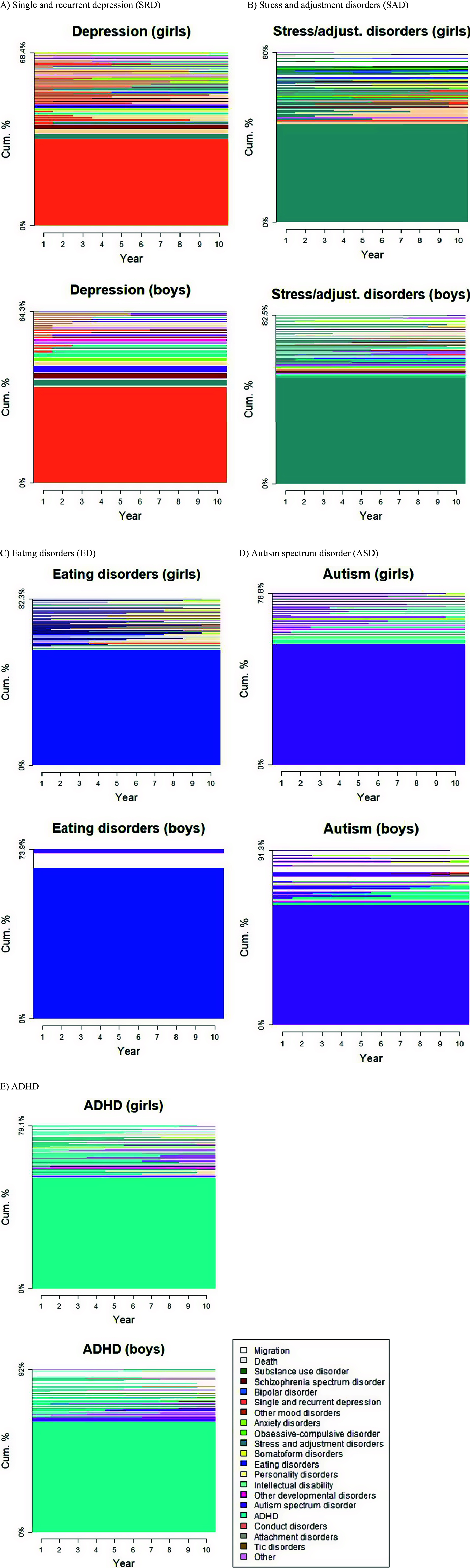
Selected contrasting sequences of diagnostic progression following an initial psychiatric diagnosis of single and recurrent depression (SRD) (A), stress and adjustment disorders (SAD) (B), eating disorders (ED) (C), autism spectrum disorder (ASD) (D), and ADHD (E). The breadth of the sequence reflects its frequency.

The state sequence analyses of the D1s of SRD, SAD, ED, ASD, and ADHD are portrayed in Figure 1 for both sexes since these diagnostic groups are major categories in terms of prevalence. Sequences for all D1 diagnoses are shown in Supplementary Figure 1.

**Figure 2.**
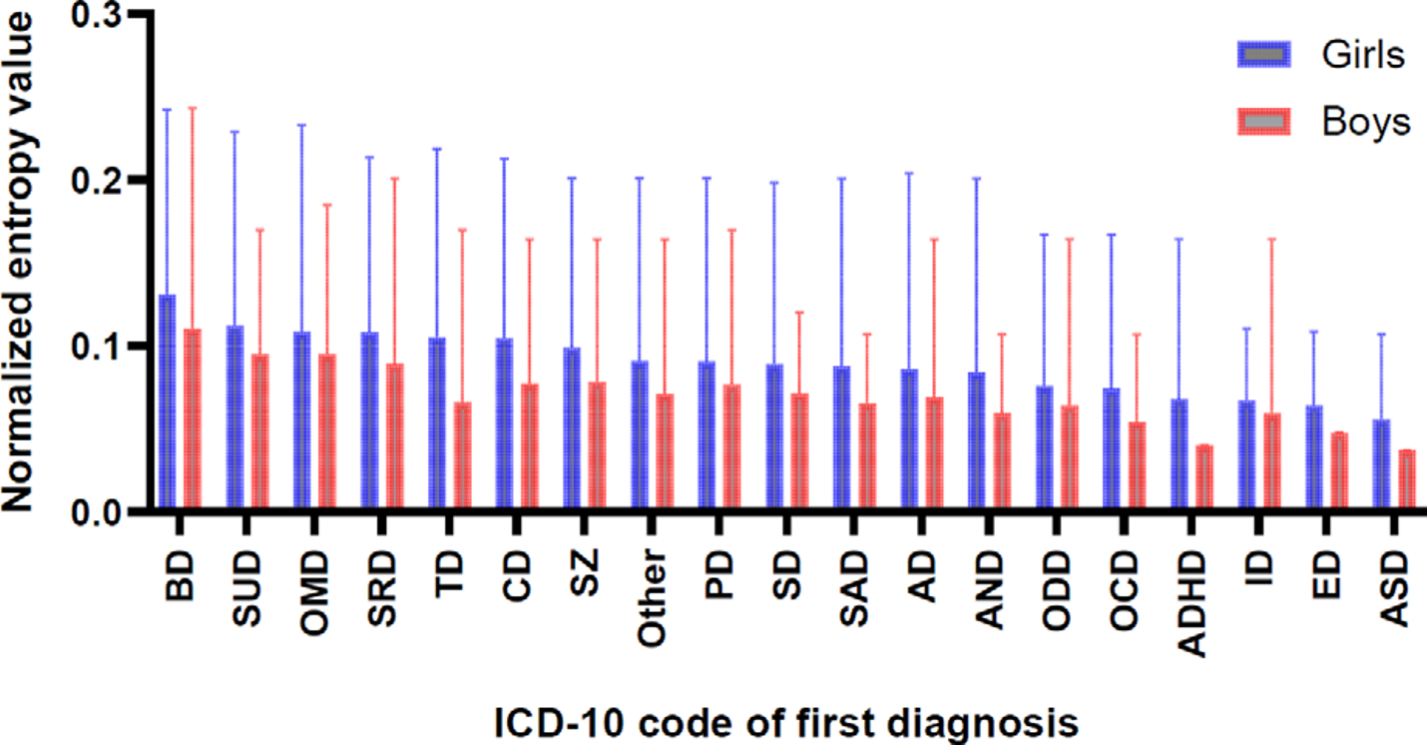
Normalized mean entropy values for sequences of subsequent diagnoses (SD) for each initial diagnosis for boys and girls separately. BD, bipolar disorder (F30-31); SUD, substance use disorder (F10-F19); SRD, single and recurrent depression (F32+F33); TD, Tic disorders (F95 (F95)); SZ, Schizophrenia spectrum disorder (F20-29); CD, conduct disorders (F91); OMD, other mood disorders (F34-F39); PD, personality disorders (F60-69), other (F99 (F51-F59, F84.2-F84.4, F88, F89, F92, F98.0-F98.6, F98.9, F99.9); SD, Somatoform disorders (F44-F48); SAD, stress and adjustment disorders (F43); AD, attachment disorder (F94 (minus F94.0)); AND, anxiety disorders (F40+F41+F93); OCD, obsessive-compulsive disorder (F42); ODD, other developmental disorders (F80-83); ADHD, attention deficit hyperactivity disorder (F90 (F90 + F98.8)); ID, intellectual disability (F70-79); ED, eating disorders (F50); ASD, autism spectrum disorder (F84 (minus F84.2-F84.4)). *p*-values for difference in mean entropy between boys and girls: F10, 0.074; F20, <0.001; F30, 0.414; F32, <0.001; F34, 0.430; F40, <0.001; F42, <0.001; F43, < 0.001; F44, 0.158; F50, 0.005; F60, 0.019; F70, 0.063; F80, 0.007; F84, <0.001; F90, <0.001; F91:< 0.001; F94, <0.001; F95, <0.001; F99, 0.005.

Overall, mean entropy values tended to be higher in diagnoses, which typically present in adolescence rather than in childhood. Thus, for example, lower entropy was observed in both sexes for ADHD and ASD presenting between ages 0 and 10 compared with ages 11–17, and for girls, this was also the case for ODD, intellectual disability (ID), SAD, and anxiety disorders (AND) (Supplementary Table 1). Overall, mean entropy values tended to be higher for girls than for boys, with significant differences for the categories F20, F32, F40, F42, F43, F50, F60, F80, F84, F90, F91, F94, F95, and F99 (Figure 2), for most also after sensitivity analyses excluding SAD as a diagnosis (Supplementary Figure 2). For girls, the highest mean entropy values were identified for the D1 of BD (please note that the majority of entropy measures are not shown due to few observations in sequences [<5]), SUD, and SRD, with means between 0.11 and 0.13. The lowest entropy values in girls were identified for the D1 of tic disorder, ASD, and ED, with means between 0.01 and 0.06 (Figure 2 and Supplementary Table 2a). Highest entropy values in boys were identified for the D1 of BD, OMDs, and SUD, with means between 0.09 and 0.11. Lowest mean entropy values in boys were identified for the D1 of tic disorder, autism, and ADHD, with means between 0.02 and 0.04 (Figure 2 and Supplementary Table 2b).

Regarding highest parental educational attainment (Supplementary Table 3a), we observed significantly higher mean entropy for girls with single or recurrent depression (SRD), OMD, SAD, ED, ID and other and higher mean entropy for boys with BD, anxiety (AND), SAD, ASD and other if the parents had short or medium compared to long educational attainment. Higher entropy was observed across several diagnostic categories in more recent years compared to the first years observed, especially for girls, with the exception of tic disorders where entropy had decreased (Supplementary Table 3b).

### State sequence analyses

Looking at the major diagnostic groups, here highlighting depression, adjustment disorder, ED, autism, and ADHD, the sequence analyses showed that for girls with a D1 of SRD, 41.0% did not change category, and the most common SDs were personality disorder (7.6%), and SAD (6.1%). For boys with a D1 of SRD, 43.0% did not change category, and the most common SDs were SAD and ADHD (3.3% for both). For girls with a D1 of SAD, 55.1% did not change category, and the most common SDs were personality disorder (6.6%) and SRD (5.0%). In boys, 62.3% did not change category, and the most common SDs were ADHD (4.6%) and schizophrenia (2.4%). For girls with at D1 of ED, 68.3% did not change category. The most common SDs were personality disorder (3.8%) and SAD (2.8%). For boys with a D1 of ED, 72.0% did not change category. The most common SD was autism (1.8%) while other shifts were too unique to be included. For girls with a D1 of autism, 66.1% did not change category. The most common SDs were ADHD (5.0%) and ID (1.81%). For boys with a D1 of autism, 74.9% did not change category. The most common SDs were ADHD (6.7%) and ID (1.7%). For girls with ADHD as their D1, 64.3% had no SD, and the most common SDs were ASD (3.8%) and personality disorder (3.3%). For boys with ADHD, 74.9% did not change category, and the most common SDs were ASD (4.3%) and SAD (1.3%) (Supplementary Table 4a and b).

### Cox regression analyses of associations between first psychiatric diagnosis and subsequent diagnoses

Figure 3A and B shows the Cox proportional hazard models for the associations between first psychiatric diagnosis (D1) and change to SDs during 10 years of follow-up for boys and girls, separately. We here highlight the hazard ratios of the abovementioned defined major categories. For girls and boys with a D1 of SRD, the highest significant HRs were identified for a SD of schizophrenia and personality disorder. For girls with a D1 of SAD, the highest significant HRs were identified for a SD of BD and schizophrenia. For boys with a D1 of SAD, the highest significant HRs were identified for a SD of personality disorder and SUD. For girls with a D1 of ED, the highest significant HRs were identified for a SD of personality disorder and schizophrenia. For boys with a D1 of ED, the highest significant HRs were identified for a SD of BD. For both girls and boys with a D1 of ASD, the highest significant HRs were identified for a SD of ID and schizophrenia. For girls with a D1 of ADHD, the highest significant HRs were identified for a SD of c and of SUD. For boys with a D1 of ADHD, the highest significant HRs were identified for a SD of SUD and a SD of ID. Overall, the highest HRs for boys were identified for a D1 of mental disorders not otherwise specified and a SD of organic disorder, for a D1 of SUD and a SD of schizophrenia, for a D1 of BD and a SD of schizophrenia, and for a D1 of schizophrenia and a SD of SUD. In girls, the highest HRs were identified for a D1 of mental disorder not otherwise specified and a SD of organic mental disorder and an SD of behavioral and emotional disorder, for a D1 of personality disorder and a SD of SUD and schizophrenia, and for a D1 of conduct disorder and a SD of later SUD (Figure 3A and B).

**Figure 3A.**
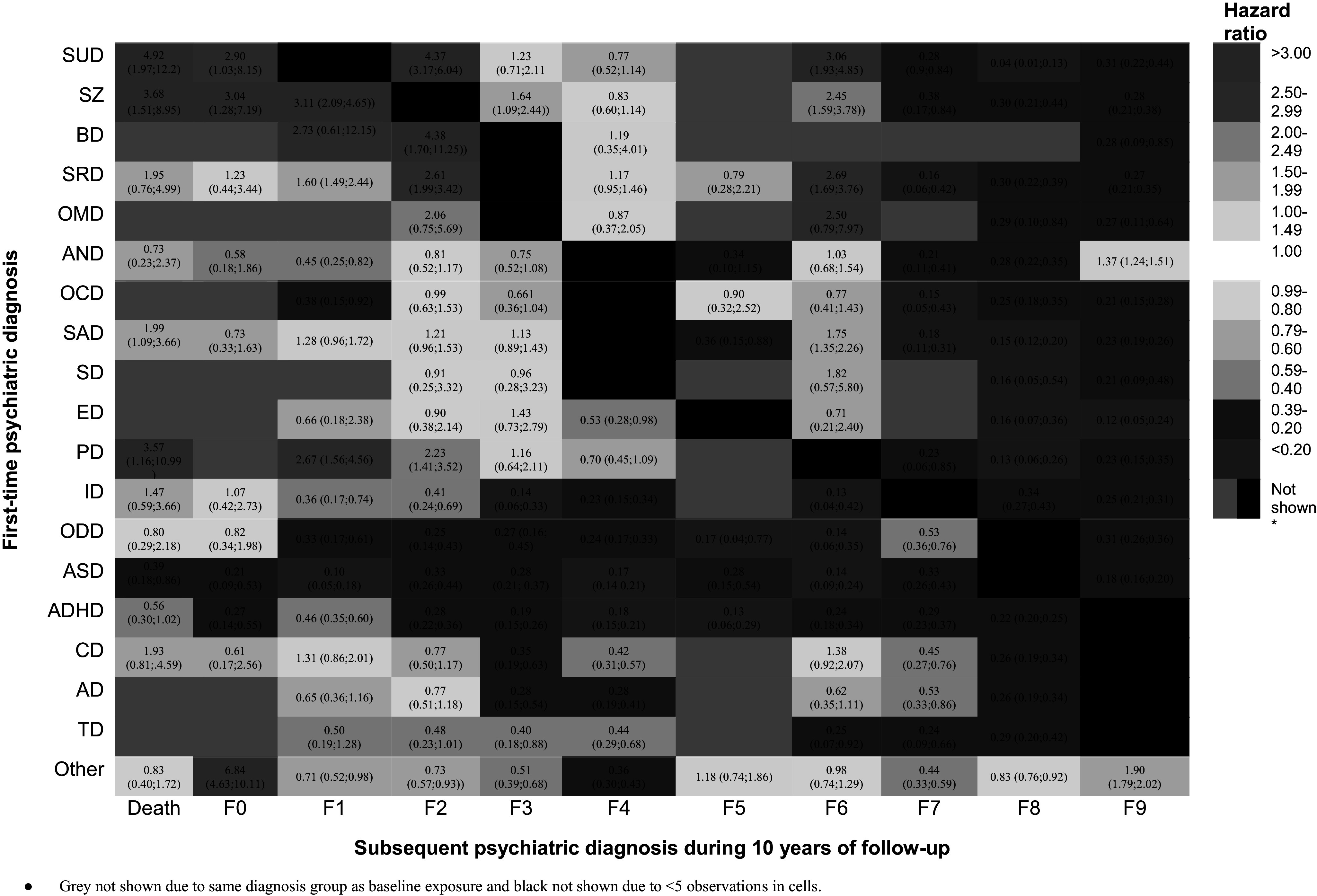
Hazard ratio estimates with 99.9% confidence intervals of the associations between primary diagnosis (D1) and subsequent diagnoses (SD) compared to the rest of the primary diagnosis (D1) estimated in 44,731 boys. SUD, substance use disorder (F10-F19); SZ, Schizophrenia spectrum disorder (F20-29); BD, bipolar disorder (F30-31); SRD, single and recurrent depression (F32+F33); OMD, other mood disorders (F34-F39); AND, anxiety disorders (F40+F41+F93); OCD, obsessive-compulsive disorder (F42); SAD, stress and adjustment disorders (F43); SD, Somatoform disorders (F44-F48); ED, eating disorders (F50); PD, personality disorders (F60-69); ID, intellectual disability (F70-79); ODD, other developmental disorders (F80-83); ASD, autism spectrum disorder (F84 (minus F84.2-F84.4)); ADHD, attention deficit hyperactivity disorder (F90 (F90 + F98.8)); CD, conduct disorders (F91); AD, attachment disorder (F94 (minus F94.0)); TD, Tic disorders (F95 (F95)), other (F99 (F51-F59, F84.2-F84.4, F88, F89, F92, F98.0-F98.6, F98.9, F99.9).

**Figure 3B.**
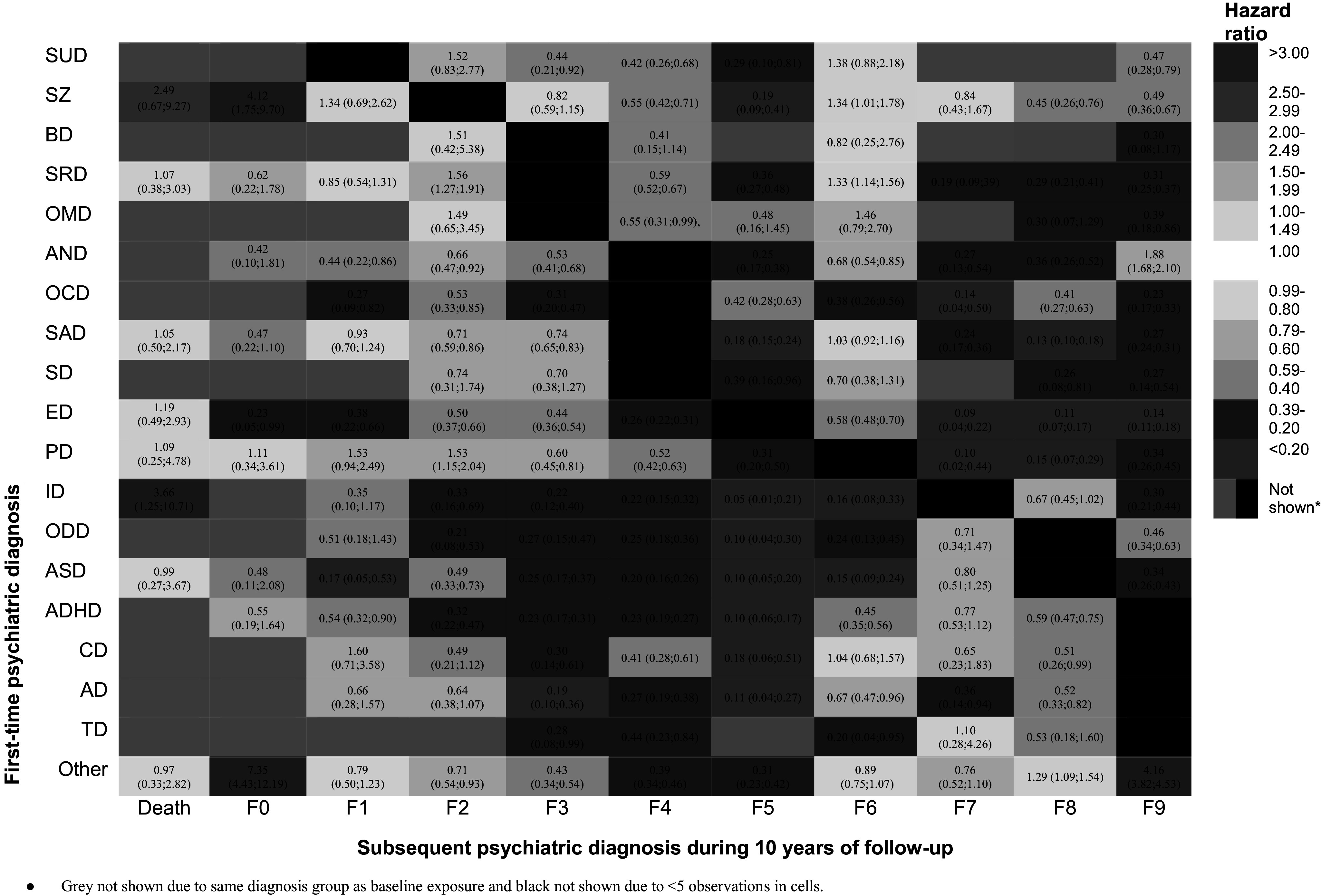
Hazard ratio estimates with 99.9% confidence intervals of the associations between primary diagnosis (D1) and subsequent diagnoses (SD) compared to the rest of the primary diagnosis (D1) estimated in 32,733 girls. SUD, substance use disorder (F10-F19); SZ, Schizophrenia spectrum disorder (F20-29); BD, bipolar disorder (F30-31); SRD, single and recurrent depression (F32+F33); OMD, other mood disorders (F34-F39); AND, anxiety disorders (F40+F41+F93); OCD, obsessive-compulsive disorder (F42); SAD, stress and adjustment disorders (F43); SD, Somatoform disorders (F44-F48); ED, eating disorders (F50); PD, personality disorders (F60-69); ID, intellectual disability (F70-79); ODD, other developmental disorders (F80-83); ASD, autism spectrum disorder (F84 (minus F84.2-F84.4)); ADHD, attention deficit hyperactivity disorder (F90 (F90 + F98.8)); CD, conduct disorders (F91); AD, attachment disorder (F94 (minus F94.0)); TD, Tic disorders (F95 (F95)), other (F99 (F51-F59, F84.2-F84.4, F88, F89, F92, F98.0-F98.6, F98.9, F99.9).

## Discussion

SAD, ED, ADHD, ASD, SRD, and ODD were the found most prevalent first-time main diagnoses across the sexes. Further, also in line with a previous study of the Danish population by Dalsgaard et al., ED and SRD were more prevalent in girls, and ASD and ODD were more prevalent in boys [4]. In line with widely accepted evidence for age-dependent patterns as for example reported by Paus, Solmi, and colleagues, we observed age-dependent patterns in onset of diagnosis, where neurodevelopmental disorders typically presented in childhood and affective disorders in adolescence [20, 21]. We also found earlier detection and diagnosis among boys than girls across major child and adolescent mental disorders. This is also in line with previous studies, for example, concerning autism, ADHD, mood, anxiety, and ED [4, 22].

We found that 46.7% of girls and 37.6% of boys had at least one diagnostic shift during 10 years of follow-up. The prevalences of overall diagnostic shifts are only partially comparable with previous studies, as these had shorter follow-up – thus, diagnostic adjustment was identified in 19% of cases in a British study of a clinical sample of children and adolescents (average age at first diagnosis = 12.24) at follow-up after 30 days [11]. Noteworthy, a possible reason for the high frequency of at least one diagnostic shift could be that registration is well integrated in Danish clinical IT systems, leading to easy and frequent updating of diagnostic register information; this could in some cases lead to, for example, the registration of a SAD as a relevant main diagnosis in an emergency ward for a patient already diagnosed with ASD [13]. Further, trends toward higher entropy in more recent years may reflect more frequent use of the child and adolescent psychiatric services as compared to previous practices.

For 12 diagnostic categories, we found statistically significant higher average entropy (diagnostic instability) for girls, compared with boys, while no categories showed higher average entropy in boys compared with girls. Similar findings of higher diagnostic instability in girls compared with boys have been indicated in previous studies, although another study did not find any differences [7]. Our findings of higher entropy among girls might reflect the later onset of diagnosis compared with boys since diagnoses with an early onset typically shows higher stability in our study, in line with the literature [7]. Further, it might reflect the higher prevalence of internalizing problems among girls in our sample, in line with previous studies, as such problems have been shown to be associated with lower diagnostic stability [8, 23]. While we cannot know if the higher entropies among girls reflect inadequate initial diagnostic conclusions or is rather an expression of truly new diagnostic conditions, the differential patterns as compared to boys call for a focus on timely detection of both first and subsequent diagnoses. Given the type of diagnostic categories where higher entropy was identified in girls diagnosed at ages 11–17 compared to at ages 0–10, including neurodevelopmental disorders and ID, findings could indicate that girls with such disorders may compensate longer but also develop more comorbidity.

We found that the highest prevalence of SDs and highest entropy values were seen among first-time main diagnoses (D1s) often presenting in adolescence such as affective disorders, psychotic illnesses, and personality disorders, while neurodevelopmental disorders, somatoform disorders, and ED had lower prevalence of secondary diagnoses (SD) and lower stability (mean entropy). Regarding diagnostic stability in specific categories, our findings of high diagnostic stability after a first diagnosis of ADHD, ASD, ID, or ED are in line with previous studies, which also found high diagnostic stability for neurodevelopmental disorders and learning disability [7]. As in our sample, Girela-Serrano et al. also found common shifts within the neurodevelopmental categories, such as shifts from autism to ADHD [7]. Further, one previous study found higher stability related to externalizing problems, especially a diagnosis of ADHD, compared to internalizing problems [8]. Our findings are partly in line with these, as we, too, found high diagnostic stability following an ADHD diagnosis as compared with diagnoses comprising internalizing problems such as depression and anxiety. This might indicate a smaller need for diagnostic adjustment in the health care sector after a first diagnosis of a neurodevelopmental disorder, as compared to other, e.g. internalizing, diagnostic categories. Our findings of lower diagnostic stabilities after a first diagnosis of mood disorder, personality disorder, schizophrenia, BD, and depression are also in line with previous findings [8, 24]. For example, lower stability of these diagnoses (in contrast to ADHD) has previously been found for a first diagnosis of anxiety and depression, and even lower stability has been found for psychotic symptoms occurring in childhood and followed up in adolescence [8, 24].

For both sexes, mental disorders not otherwise specified were associated with higher hazards of subsequent organic disorders. This is not surprising since organic disorders present with differential symptom patterns, which would most likely cause the child and adolescent psychiatrist to seek further investigation upon a first diagnostic conclusion. It highlights that when symptoms do not fit well with the main psychiatric diagnostic categories, the risk of underlying organic disorder is higher.

### Strengths and limitations

The data source is a great strength because the Danish nationwide health care registers constitute optimal and unique conditions to examine psychiatric trajectories across all major diagnostic categories presenting in childhood and adolescence during the full age range from 0 to 17 years across several decades with minimal loss to follow-up [13, 14, 25]. Separate analyses across the sexes and social backgrounds further cover literature gaps.

Small sample sizes limited the analyses of BD. Further, our analyses only encapsule shifts in main diagnoses. To fit the data within the sequence analyses, the analyses were limited to only include the last diagnosis in each of the 6 months intervals investigated over the course of 10 years. Thus, the entropy measures can be interpreted as diagnostic variability based on aggregated information in these 6 months periods over a course of 10 years. Finally, death, although very rare in this young population, is a competing risk for receiving other diagnoses.

## Conclusions

Diagnostic shifts following a first diagnosis of mental disorder in childhood or adolescence are common. Diagnostic stability assessed within a 10-year follow-up is higher for diagnoses with a mean age at onset in childhood as compared to adolescence, indicating more complexity and higher help-seeking in the health care sector. Girls have lower diagnostic stability than boys. Findings of adolescent diagnostic instability, particularly concerning affective or psychotic disorders, highlight the need for close collaboration across adolescent and adult psychiatric services.

## Supporting information

10.1192/j.eurpsy.2025.10091.sm001 Krantz et al. supplementary material Krantz et al. supplementary material

## Data Availability

The data that support the findings of this study are available from Statistics Denmark. However, restrictions apply to the availability of the data that were used under license for this study. Contact information for data requests: BFH-FP-CKFF-Projektdatabasen <ckffprojektdatabasen.bispebjerg-frederiksberg-hospitaler@regionh.dk>).

## Supplementary material

The supplementary material for this article can be found at http://doi.org/10.1192/j.eurpsy.2025.10091.
