# Supplementary material for: Diagnostic trajectories and stability of mental disorders in childhood and adolescence – A nation-wide cohort study using sequence analysis
Source: Eur Psychiatry. 2025 Aug 26;68(1):e126. doi: 10.1192/j.eurpsy.2025.10091 (PMC12438990; doi:10.1192/j.eurpsy.2025.10091)
Supplement: Krantz et al. supplementary material [file S0924933825100916sup001.docx]

**Supplementary tables and figures**

**Supplementary Figure 1:** State sequence analyses


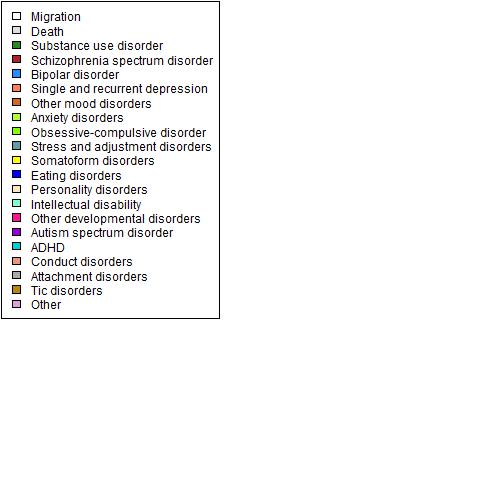


| 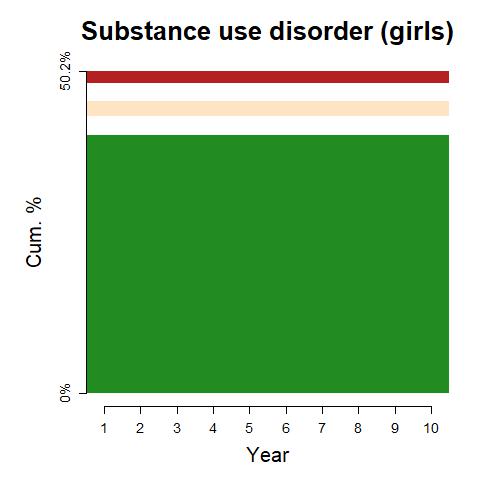 | 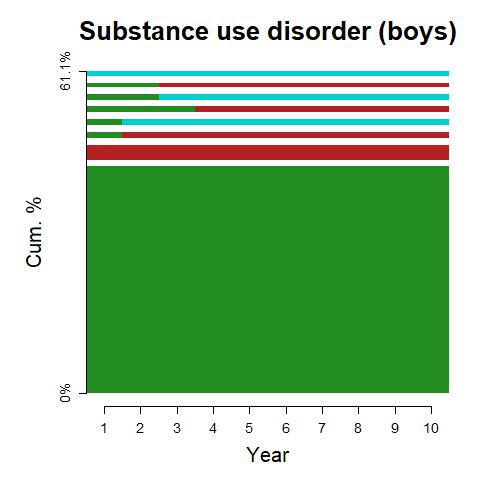 |
| --- | --- |
| 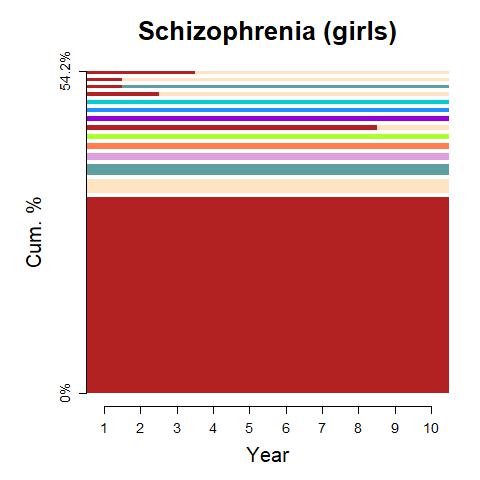 | 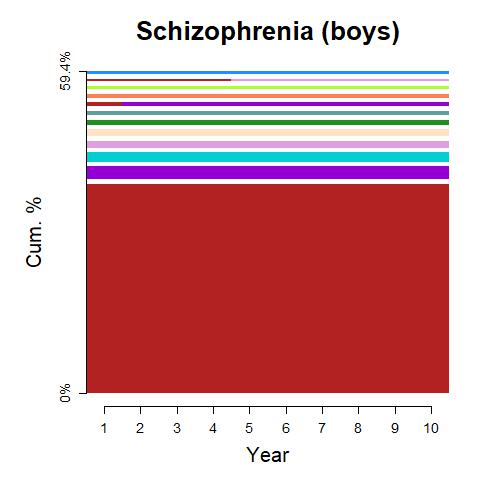 |

**Figure S1a** Sequence analyses of subsequent diagnoses (SD) after all included first-time main psychiatric diagnosis (D1) during 10 years of follow-up. The breadth of the sequence reflects its frequency. Please note that the cumulative percent differs among D1s due to different number of sequences below the threshold (see Methods)

.

| 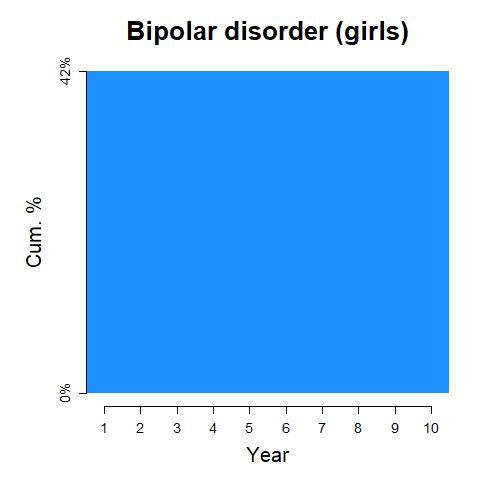 | 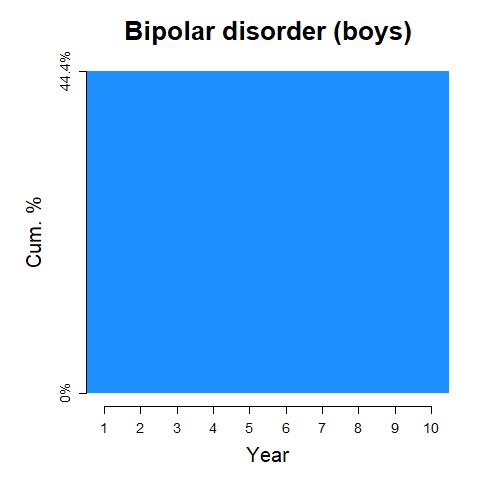 |
| --- | --- |
| 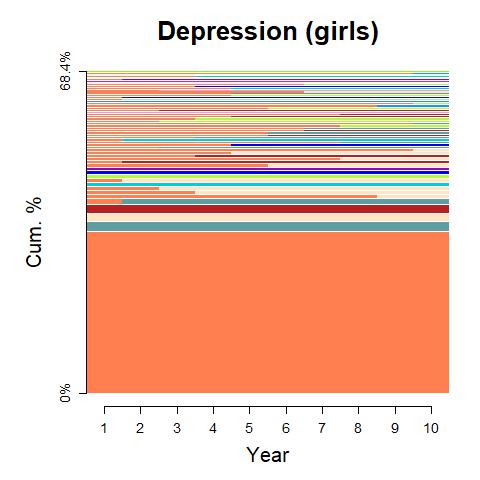 | 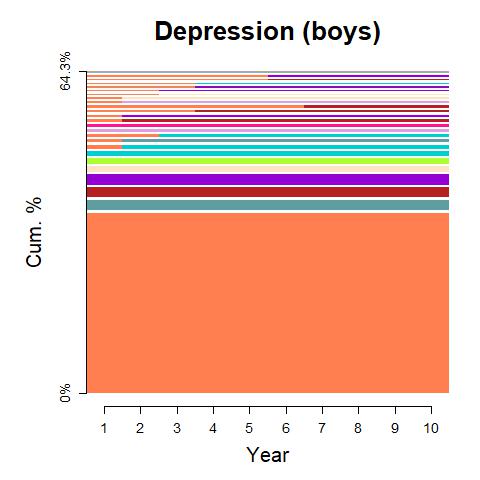 |

**Figure S1b** Sequence analyses of subsequent diagnoses (SD) after all included first-time main psychiatric diagnosis (D1) during 10 years of follow-up. The breadth of the sequence reflects its frequency. Please note that the cumulative percent differs among D1s due to different number of sequences below the threshold (see Methods).

| 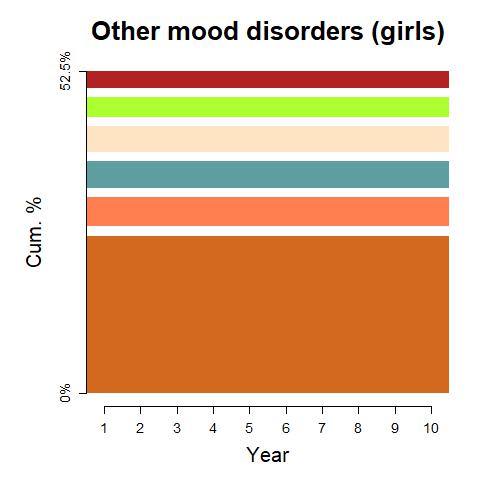 | 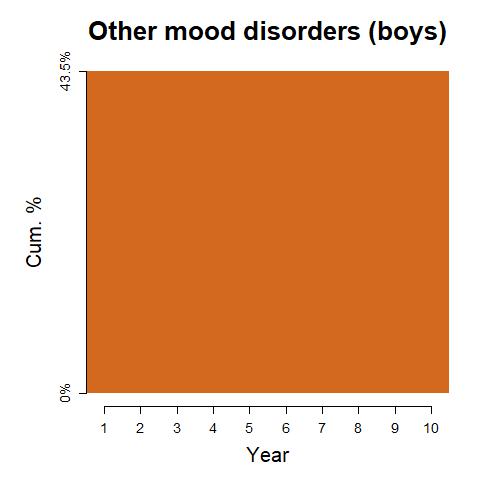 |
| --- | --- |
| 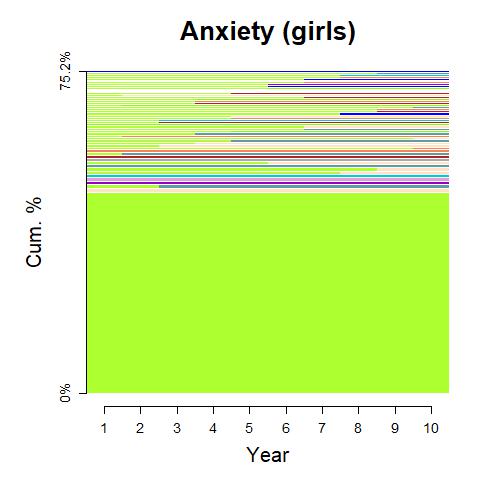 | 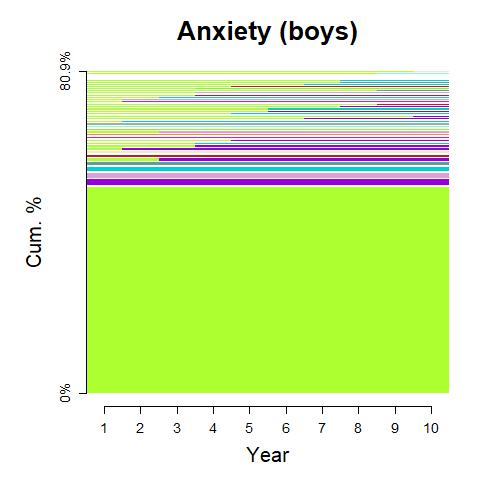 |

**Figure S1c** Sequence analyses of subsequent diagnoses (SD) after all included first-time main psychiatric diagnosis (D1) during 10 years of follow-up. The breadth of the sequence reflects its frequency. Please note that the cumulative percent differs among D1s due to different number of sequences below the threshold (see Methods).

| 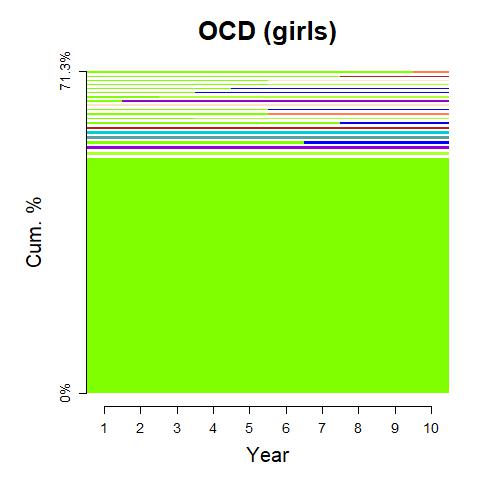 | 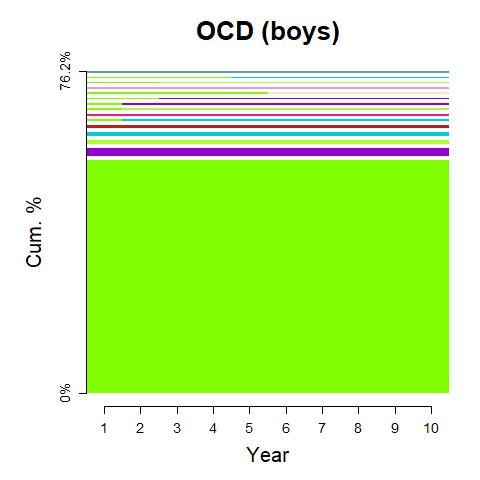 |
| --- | --- |
| 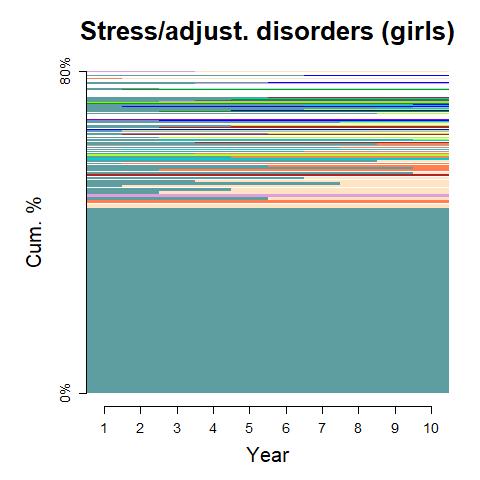 | 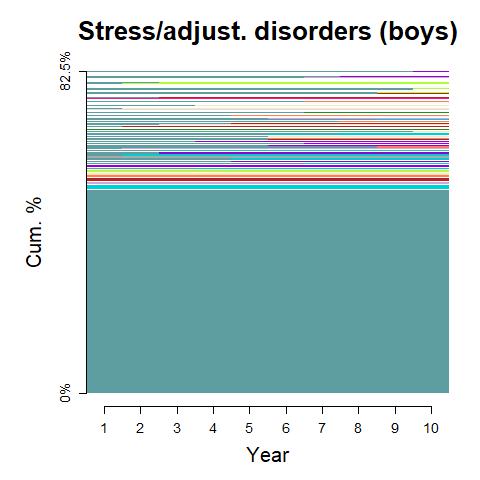 |

**Figure S1d** Sequence analyses of subsequent diagnoses (SD) after all included first-time main psychiatric diagnosis (D1) during 10 years of follow-up. The breadth of the sequence reflects its frequency. Please note that the cumulative percent differs among D1s due to different number of sequences below the threshold (see Methods).

| 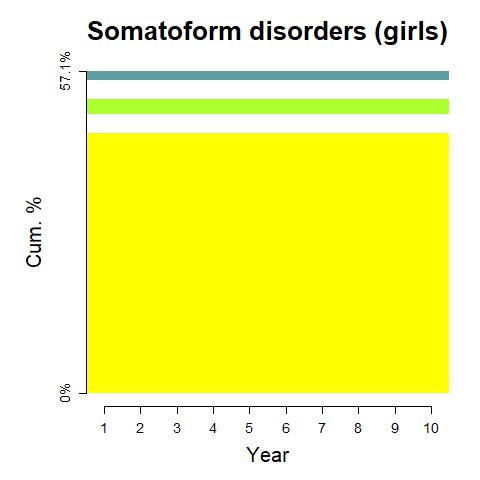 | 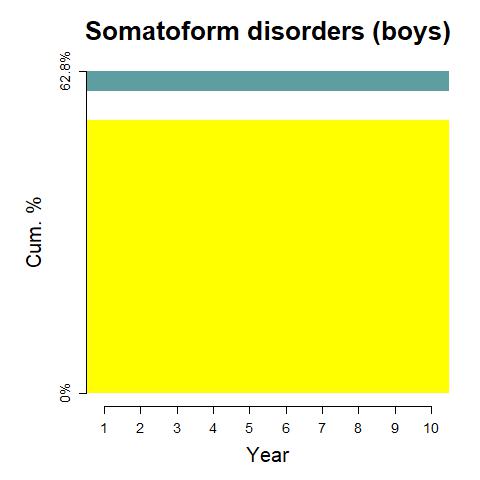 |
| --- | --- |
| 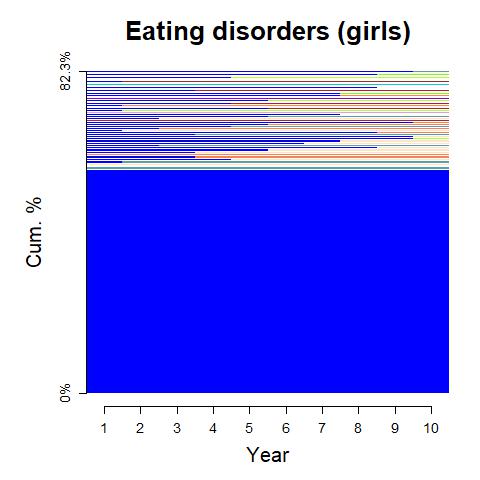 | 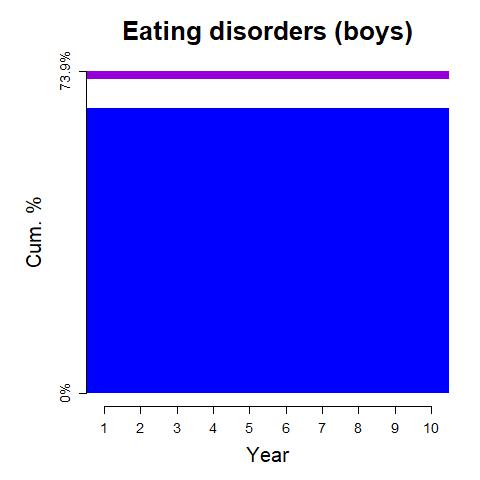 |

**Figure S1e** Sequence analyses of subsequent diagnoses (SD) after all included first-time main psychiatric diagnosis (D1) during 10 years of follow-up. The breadth of the sequence reflects its frequency. Please note that the cumulative percent differs among D1s due to different number of sequences below the threshold (see Methods).

| 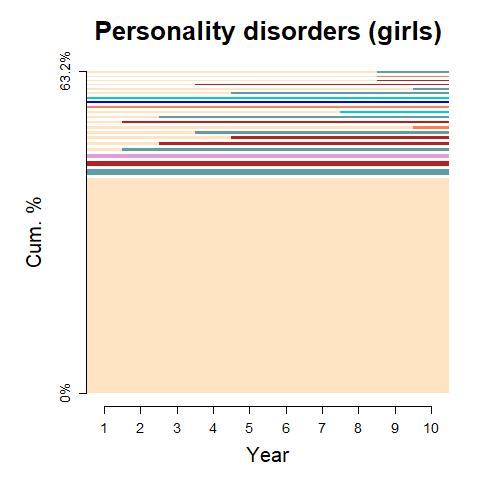 | 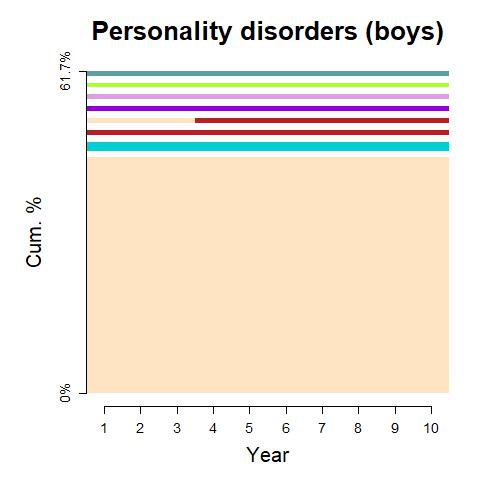 |
| --- | --- |
| 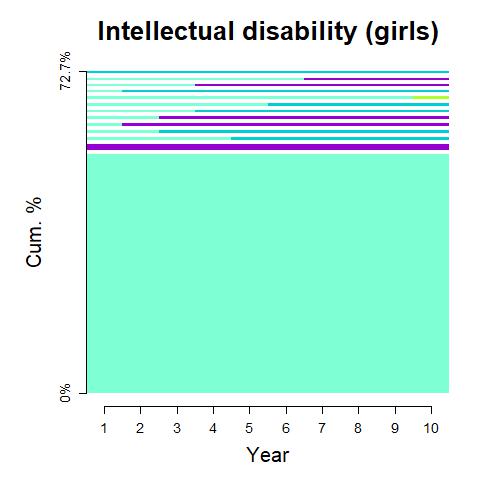 | 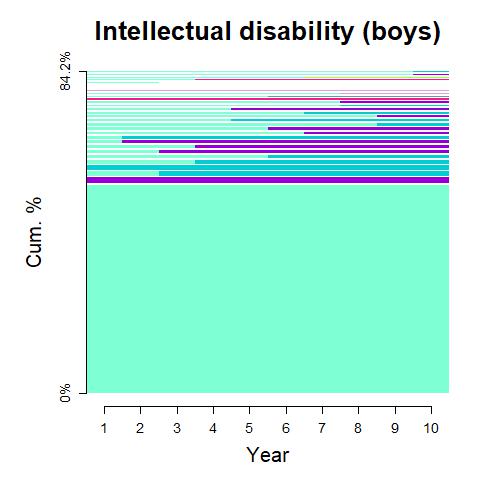 |

**Figure S1f** Sequence analyses of subsequent diagnoses (SD) after all included first-time main psychiatric diagnosis (D1) during 10 years of follow-up. The breadth of the sequence reflects its frequency. Please note that the cumulative percent differs among D1s due to different number of sequences below the threshold (see Methods).

| 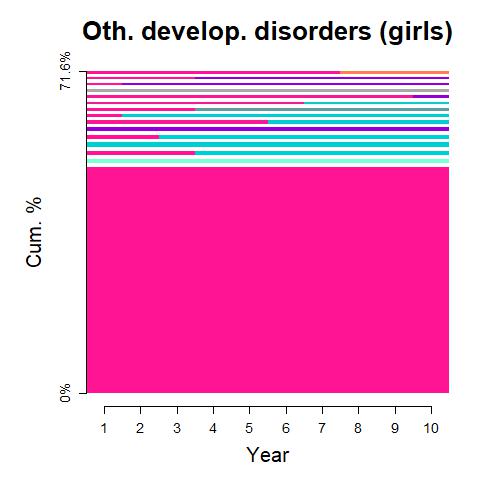 | 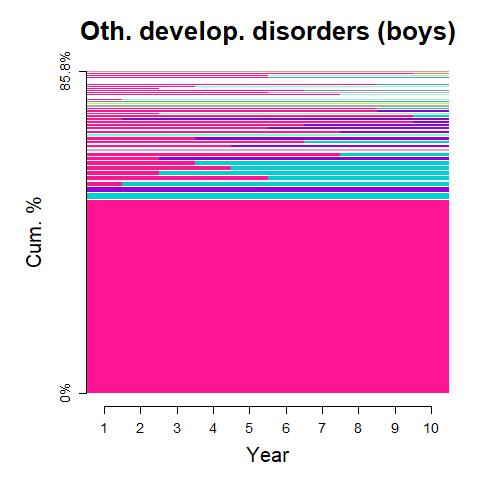 |
| --- | --- |
| 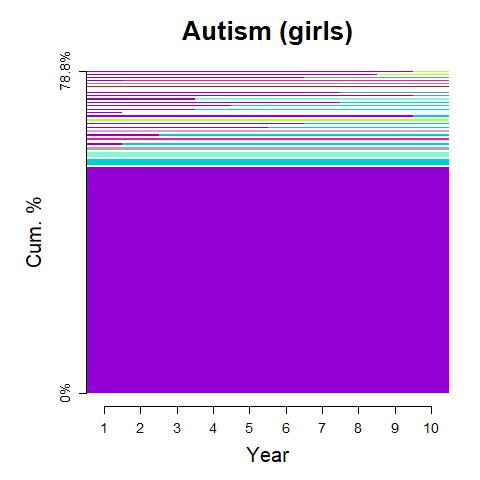 | 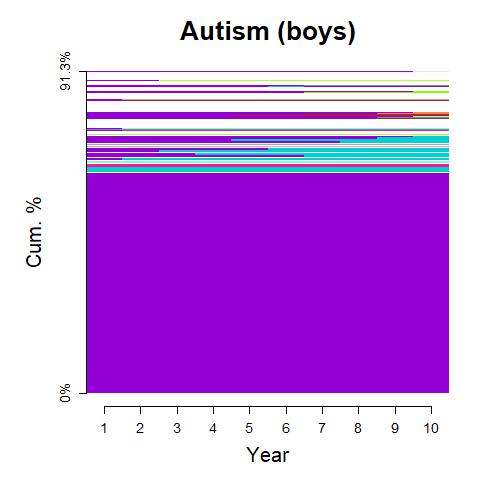 |

**Figure S1g** Sequence analyses of subsequent diagnoses (SD) after all included first-time main psychiatric diagnosis (D1) during 10 years of follow-up. The breadth of the sequence reflects its frequency. Please note that the cumulative percent differs among D1s due to different number of sequences below the threshold (see Methods).

| 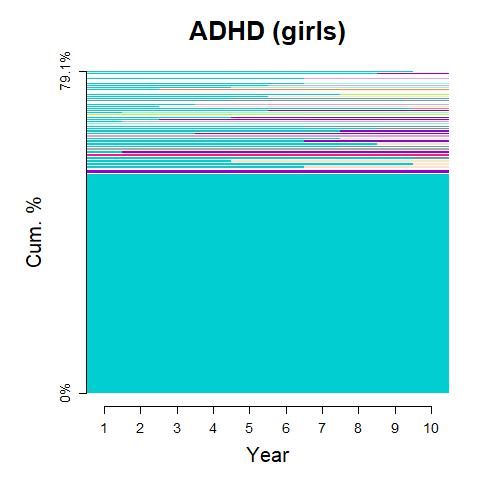 | 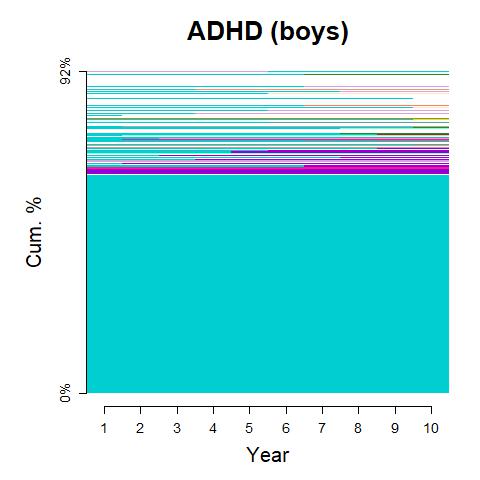 |
| --- | --- |
| 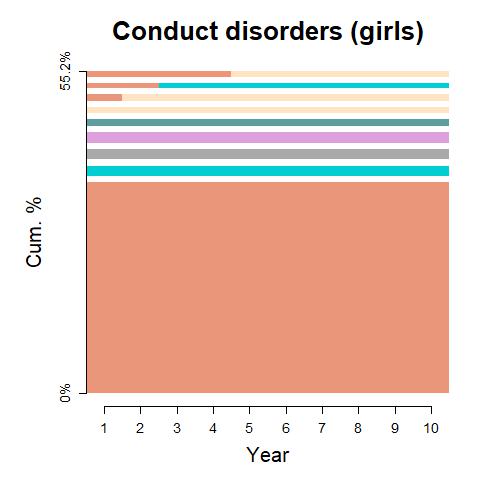 | 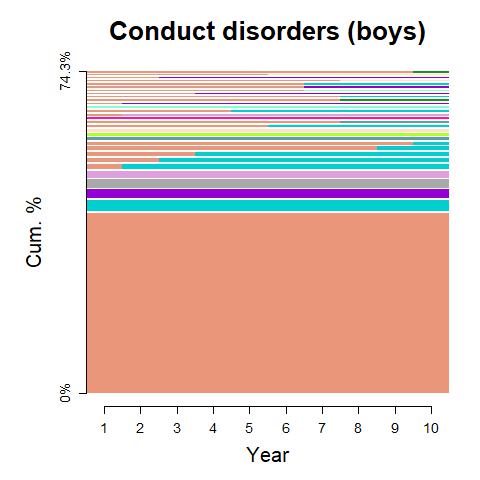 |

**Figure S1h** Sequence analyses of subsequent diagnoses (SD) after all included first-time main psychiatric diagnosis (D1) during 10 years of follow-up. The breadth of the sequence reflects its frequency. Please note that the cumulative percent differs among D1s due to different number of sequences below the threshold (see Methods).

| 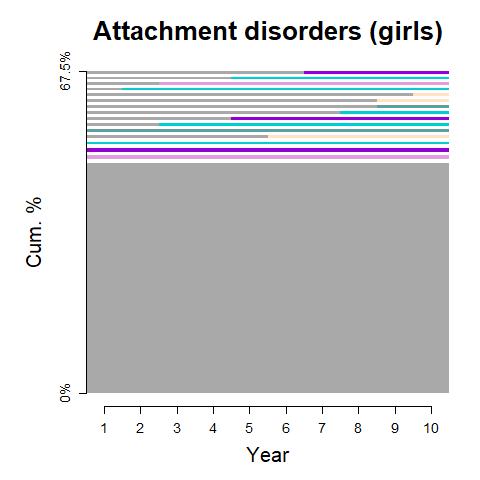 | 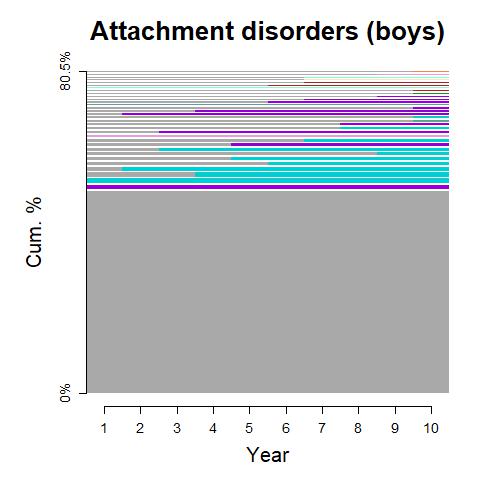 |
| --- | --- |
| 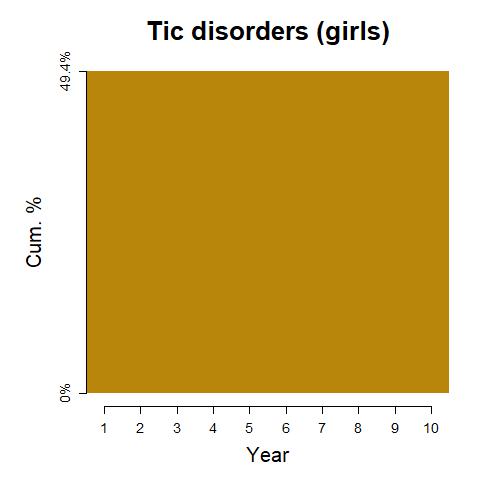 | 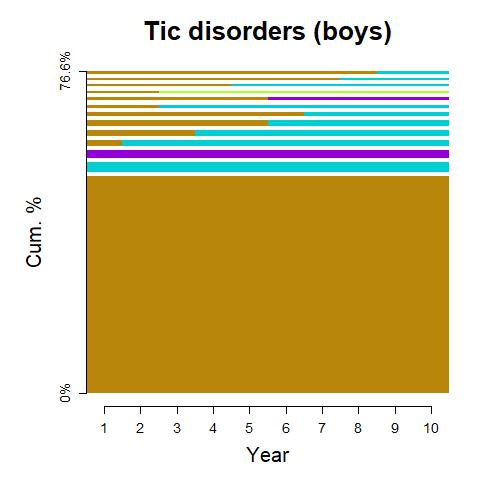 |

**Figure S1i** Sequence analyses of subsequent diagnoses (SD) after all included first-time main psychiatric diagnosis (D1) during 10 years of follow-up. The breadth of the sequence reflects its frequency. Please note that the cumulative percent differs among D1s due to different number of sequences below the threshold (see Methods).

| 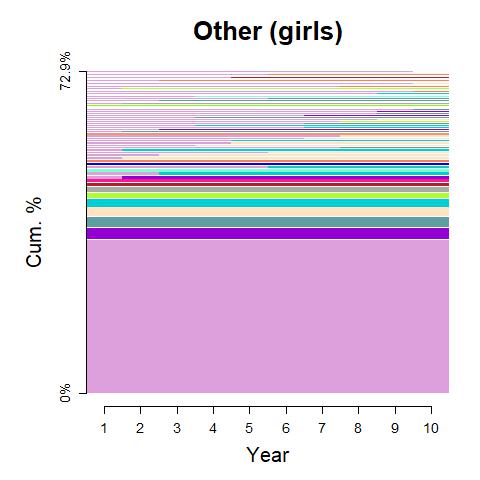 | 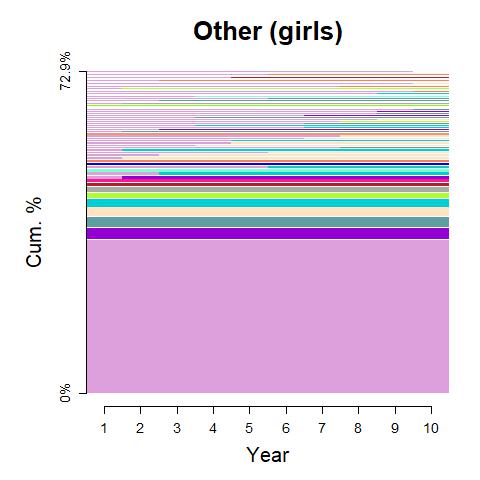 |
| --- | --- |
|  |  |

**Figure S1je** Sequence analyses of subsequent diagnoses (SD) after all included first-time main psychiatric diagnosis (D1) during 10 years of follow-up. The breadth of the sequence reflects its frequency. Please note that the cumulative percent differs among D1s due to different number of sequences below the threshold (see Methods).

**Supplementary Table 1** Mean entropy for age 0-10 and 11-17 years for boys and girls

|  | Girls | | | Boys | | |
| --- | --- | --- | --- | --- | --- | --- |
|  | 0-10 years | 11-17 years |  | 0-10 years | 11-17 years |  |
|  | Mean entropy | | p-values | Mean entropy | | p-values |
| **Substance use disorder (SUD**) | - | - | - | - | - | - |
| **Schizophrenia spectrum disorder (SZ)** | 0.092 | 0.099 | 0.751 | 0.089 | 0.078 | 0.475 |
| **Bipolar disorder (BD)** | - | - | - | - | - | - |
| **Single and recurrent depression (SRD)** | 0.133 | 0.108 | 0.186 | 0.092 | 0.089 | 0.780 |
| **Other mood disorders (OMD)** | 0.167 | 0.110 | 0.277 | 0.110 | 0.098 | 0.780 |
| **Anxiety disorders (AND)** | 0.066 | 0.091 | **<0.001** | 0.059 | 0.060 | 0.847 |
| **Obsessive-compulsive disorder (OCD)** | 0.077 | 0.074 | 0.664 | 0.061 | 0.052 | 0.202 |
| **Stress and adjustment disorders (SAD)** | 0.042 | 0.094 | **<0.001** | 0.050 | 0.072 | **<0.001** |
| **Somatoform disorders (SD)** | 0.133 | 0.090 | 0.176 | 0.066 | 0.076 | 0.747 |
| **Eating disorders (ED)** | 0.068 | 0.064 | 0.731 | 0.078 | 0.047 | 0.229 |
| **Personality disorders (PD)** | 0.141 | 0.090 | 0.403 | 0.093 | 0.076 | 0.475 |
| **Intellectual disability (ID)** | 0.053 | 0.085 | **<0.001** | 0.065 | 0.057 | 0.109 |
| **Other developmental disorders (ODD)** | 0.061 | 0.099 | **<0.001** | 0.068 | 0.059 | 0.039 |
| **Autism spectrum disorder (ASD)** | 0.040 | 0.076 | **<0.001** | 0.037 | 0.041 | **0.042** |
| **ADHD** | 0.052 | 0.086 | **<0.001** | 0.036 | 0.045 | **<0.001** |
| **Conduct disorders (CD)** | 0.099 | 0.113 | 0.287 | 0.075 | 0.083 | 0.151 |
| **Attachment disorders (AD)** | 0.061 | 0.135 | **<0.001** | 0.064 | 0.083 | **0.001** |
| **Tic disorders (TD)** | 0.103 | 0.120 | 0.419 | 0.069 | 0.066 | 0.704 |
| **Other** | 0.062 | 0.110 | **<0.001** | 0.067 | 0.077 | **0.001** |

**Supplementary Figure 2:** Sensitivity analysis of the normalized mean entropy values for sequences of subsequent diagnoses (SD) for each initial diagnosis for boys and girls separately after exclusion of stress and adjustment disorder as a subsequent diagnosis.

**
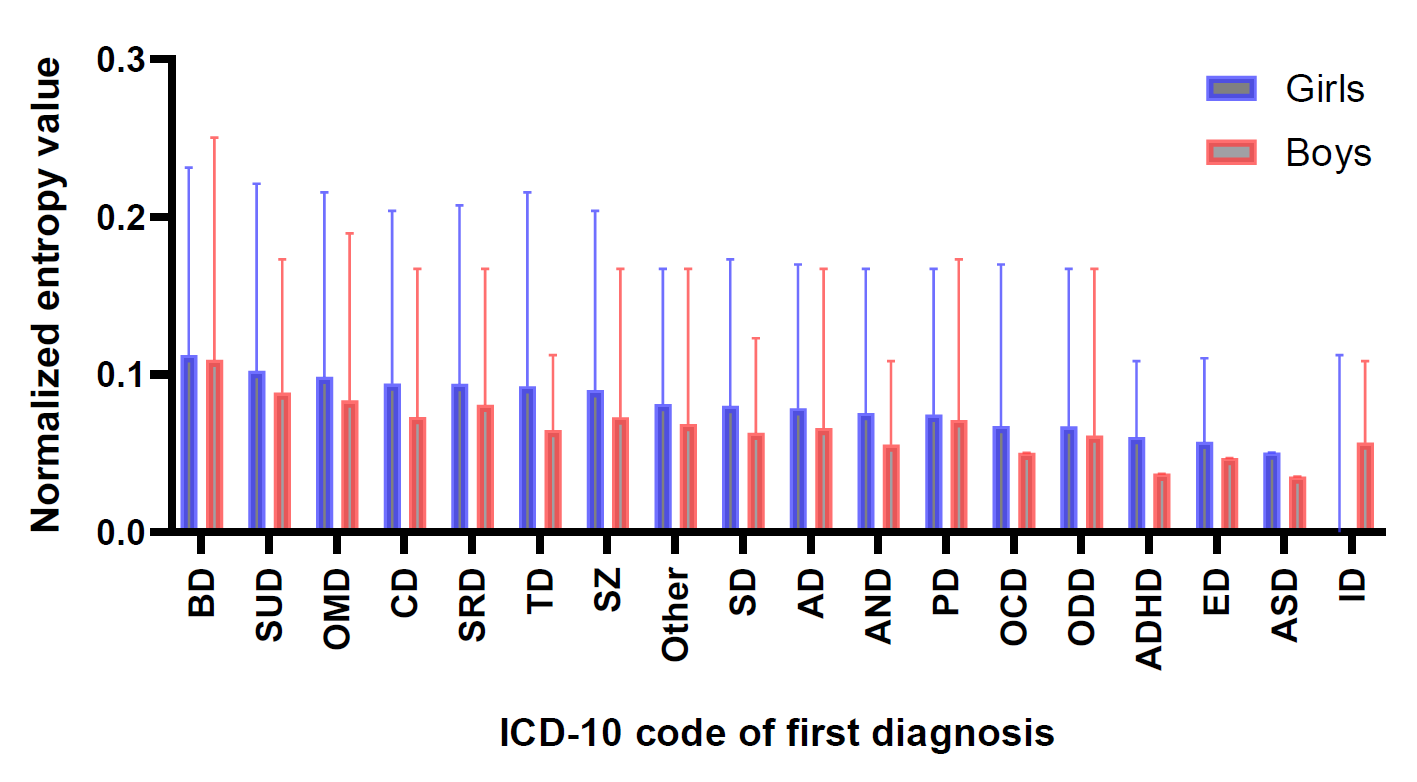
**

* p-values for difference in mean entropy between boys and girls: F10: 0.139, F20: 0.001, F30: 0.906, F32: 0.001, F34: 0.357, F40: <0.001, F42: <0.001, F44: 0.155, F50: 0.079, F60: 0.544, F70: 0.343, F80: 0.175, F84: <0.001, F90: <0.001, F91: 0.001, F94: 0.006, F95: 0.007, F99: 0.015. Compared with main analyses, girls and boys no longer differed for the categories F50, 60 and 80.

Legends: BD: Bipolar disorder (F30-31), SUD: Substance use disorder (F10-F19), SRD: Single and recurrent depression (F32+F33), TD: Tic disorders (F95 (F95)), SZ: Schizophrenia spectrum disorder (F20-29), CD: Conduct disorders (F91), OMD: Other mood disorders (F34-F39), PD: Personality disorders (F60-69), Other (F99 (F51-F59, F84.2-F84.4, F88, F89, F92, F98.0-F98.6, F98.9, F99.9), SD: Somatoform disorders (F44-F48), AD: Attachment disorder (F94 (minus F94.0)), AND: Anxiety disorders (F40+F41+F93), OCD: Obsessive-compulsive disorder (F42), ODD: Other developmental disorders (F80-83), ADHD: Attention deficit hyperactivity disorder (F90 (F90 + F98.8)), ID: Intellectual disability (F70-79), ED: Eating disorders (F50), ASD: Autism spectrum disorder (F84 (minus F84.2-F84.4)),

**Supplementary Table 2a:** Entropy measures for 1 year interval sequence analyses - girls

|  | **Mean** | **1st quartile** | **3rd quartile** |  |
| --- | --- | --- | --- | --- |
| F10 | 0.1123 | 0 | 0.2286 |  |
| F20 | 0.0992 | 0 | 0.2006 |  |
| F30 | 0.1311 | 0 | 0.2421 |  |
| F32 | 0.1085 | 0 | 0.2133 |  |
| F34 | 0.1078 | 0 | 0.2328 |  |
| F40 | 0.0841 | 0 | 0.2006 |  |
| F42 | 0.0743 | 0 | 0.1670 |  |
| F43 | 0.0879 | 0 | 0.2006 |  |
| F44 | 0.0892 | 0 | 0.1981 |  |
| F50 | 0.0641 | 0 | 0.1095 |  |
| F60 | 0.0905 | 0 | 0.2006 |  |
| F70 | 0.0672 | 0 | 0.1104 |  |
| F80 | 0.0759 | 0 | 0.1670 |  |
| F84 | 0.0557 | 0 | 0.1068 |  |
| F90 | 0.0689 | 0 | 0.1644 |  |
| F91 | 0.1050 | 0 | 0.2127 |  |
| F94 | 0.0862 | 0 | 0.2039 |  |
| F95 | 0.0154 | 0 | 0.2187 |  |
| F99 | 0.0908 | 0 | 0.2006 |  |

**Supplementary Table 2b:** Entropy measures for 1 year interval sequence analyses – boys

|  | **Mean** | **1st quartile** | **3rd quartile** |
| --- | --- | --- | --- |
| F10 | 0.0951 | 0 | 0.1700 |
| F20 | 0.0784 | 0 | 0.1644 |
| F30 | 0.1104 | 0 | 0.2432 |
| F32 | 0.0892 | 0 | 0.2006 |
| F34 | 0.0951 | 0 | 0.1848 |
| F40 | 0.0593 | 0 | 0.1068 |
| F42 | 0.0543 | 0 | 0.1068 |
| F43 | 0.0654 | 0 | 0.1068 |
| F44 | 0.0716 | 0 | 0.1200 |
| F50 | 0.0477 | 0 | 0.0000 |
| F60 | 0.0768 | 0 | 0.16995 |
| F70 | 0.0593 | 0 | 0.1644 |
| F80 | 0.0640 | 0 | 0.1644 |
| F84 | 0.0372 | 0 | 0.0000 |
| F90 | 0.0400 | 0 | 0.0000 |
| F91 | 0.0774 | 0 | 0.1644 |
| F94 | 0.0692 | 0 | 0.1644 |
| F95 | 0.0663 | 0 | 0.1700 |
| F99 | 0.0711 | 0 | 0.1644 |

**Supplementary Table 3a** Mean entropy based on highest obtained educational level among parents for boys and girls

|  | Girls | | | | Boys | | | |
| --- | --- | --- | --- | --- | --- | --- | --- | --- |
|  | Highest obtained educational level of parents* | | | | Highest obtained educational level of parents* | | | |
|  | Short | Medium | Long |  | Short | Medium | Long |  |
|  | Mean entropy | | | p-value** | Mean entropy | | | p-value** |
| **Substance use disorder (SUD**) | 0.104 | 0.125 | 0.112 | 0.550 | 0.099 | 0.100 | 0.081 | 0.260 |
| **Schizophrenia spectrum disorder (SZ)** | 0.118 | 0.096 | 0.093 | **0.047** | 0.090 | 0.084 | 0.071 | 0.067 |
| **Bipolar disorder (BD)** | 0.267 | 0.142 | 0.133 | 0.159 | 0.255 | 0.114 | 0.104 | **0.044** |
| **Single and recurrent depression (SRD)** | 0.125 | 0.105 | 0.107 | 0.087 | 0.106 | 0.089 | 0.084 | **0.048** |
| **Other mood disorders (OMD)** | 0.150 | 0.138 | 0.069 | **0.010** | 0.165 | 0.072 | 0.124 | 0.916 |
| **Anxiety disorders (AND)** | 0.082 | 0.084 | 0.085 | 0.690 | 0.070 | 0.060 | 0.051 | **0.007** |
| **Obsessive-compulsive disorder (OCD)** | 0.112 | 0.070 | 0.075 | 0.141 | 0.059 | 0.055 | 0.053 | 0.559 |
| **Stress and adjustment disorders (SAD)** | 0.095 | 0.089 | 0.082 | **0.001** | 0.074 | 0.067 | 0.055 | **>0.001** |
| **Somatoform disorders (SD)** | 0.113 | 0.094 | 0.107 | 0.949 | 0.076 | 0.075 | 0.100 | 0.426 |
| **Eating disorders (ED)** | 0.081 | 0.064 | 0.061 | **0.005** | 0.093 | 0.047 | 0.048 | 0.118 |
| **Personality disorders (PD)** | 0.100 | 0.090 | 0.090 | 0.266 | 0.086 | 0.081 | 0.071 | 0.322 |
| **Intellectual disability (ID)** | 0.072 | 0.075 | 0.049 | **0.047** | 0.067 | 0.056 | 0.064 | 0.423 |
| **Other developmental disorders (ODD)** | 0.080 | 0.076 | 0.083 | 0.721 | 0.068 | 0.063 | 0.068 | 0.854 |
| **Autism spectrum disorder (ASD)** | 0.046 | 0.058 | 0.056 | 0.402 | 0.042 | 0.038 | 0.034 | **<0.001** |
| **ADHD** | 0.070 | 0.067 | 0.069 | 0.890 | 0.045 | 0.037 | 0.040 | 0.069 |
| **Conduct disorders (CD)** | 0.120 | 0.109 | 0.094 | 0.169 | 0.085 | 0.076 | 0.077 | 0.319 |
| **Attachment disorders (AD)** | 0.089 | 0.087 | 0.085 | 0.739 | 0.071 | 0.072 | 0.068 | 0.805 |
| **Tic disorders (TD)** | 0.120 | 0.139 | 0.095 | 0.181 | 0.081 | 0.067 | 0.071 | 0.701 |
| **Other** | 0.094 | 0.096 | 0.077 | **0.003** | 0.080 | 0.069 | 0.066 | **0.002** |

**Supplementary Table 3b** Mean entropy based on calendar year for first diagnosis for boys and girls

|  | **Girls** | | | | **Boys** | | | |
| --- | --- | --- | --- | --- | --- | --- | --- | --- |
|  | **1996-2001** | **2002-2006** | **2007-2011** |  | **1996-2001** | **2002-2006** | **2007-2011** |  |
|  | **Mean entropy** | | | **p-value**** | **Mean entropy** | | | **p-value**** |
| Substance use disorder (SUD) | 0.084 | 0.126 | 0.135 | **0.027** | 0.027 | 0.109 | 0.106 | **<0.001** |
| Schizophrenia spectrum disorder (SZ) | 0.111 | 0.098 | 0.099 | 0.368 | 0.077 | 0.109 | 0.087 | 0.513 |
| Bipolar disorder (BD) | 0.100 | 0.151 | 0.176 | 0.144 | 0.169 | 0.128 | 0.117 | 0.406 |
| Single and recurrent depression (SRD) | 0.099 | 0.102 | 0.114 | **0.005** | 0.078 | 0.088 | 0.092 | 0.135 |
| Other mood disorders (OMD) | 0.112 | 0.108 | 0.140 | 0.328 | 0.086 | 0.093 | 0.120 | 0.317 |
| Anxiety disorders (AND) | 0.062 | 0.087 | 0.099 | **<0.001** | 0.049 | 0.065 | 0.066 | **0.001** |
| Obsessive-compulsive disorder (OCD) | 0.072 | 0.069 | 0.079 | 0.279 | 0.049 | 0.052 | 0.060 | 0.118 |
| Stress and adjustment disorders (SAD) | 0.063 | 0.090 | 0.100 | **<0.001** | 0.047 | 0.064 | 0.077 | **<0.001** |
| Somatoform disorders (SD) | 0.069 | 0.129 | 0.111 | **0.026** | 0.064 | 0.062 | 0.119 | **0.049** |
| Eating disorders (ED) | 0.058 | 0.061 | 0.070 | **0.003** | 0.034 | 0.069 | 0.054 | 0.257 |
| Personality disorders (PD) | 0.087 | 0.092 | 0.101 | 0.056 | 0.066 | 0.085 | 0.097 | **0.007** |
| Intellectual disability (ID) | 0.052 | 0.074 | 0.075 | **0.018** | 0.055 | 0.065 | 0.065 | 0.091 |
| Other developmental disorders (ODD) | 0.051 | 0.098 | 0.095 | **<0.001** | 0.055 | 0.073 | 0.070 | **<0.001** |
| Autism spectrum disorder (ASD) | 0.033 | 0.052 | 0.063 | **<0.001** | 0.027 | 0.042 | 0.040 | **<0.001** |
| ADHD | 0.071 | 0.069 | 0.068 | 0.679 | 0.000 | 0.041 | 0.040 | **<0.001** |
| Conduct disorders (CD) | 0.091 | 0.097 | 0.136 | **0.002** | 0.063 | 0.095 | 0.090 | **<0.001** |
| Attachment disorders (AD) | 0.061 | 0.094 | 0.107 | **<0.001** | 0.056 | 0.077 | 0.082 | **<0.001** |
| Tic disorders (TD) | 0.125 | 0.108 | 0.036 | **<0.001** | 0.049 | 0.081 | 0.071 | 0.135 |
| Other | 0.071 | 0.092 | 0.106 | **<0.001** | 0.054 | 0.080 | 0.081 | **<0.001** |

**Supplementary Table 4a:** Exact numbers and percentages in the sequence analyses for girls

The number before the slash is the diagnostic category and the number after the slash is the number of 6-month periods spend in that category. For example, “10/20” means a sequence of 20 periods in the category F10: schizophrenia and “10/1-43/19” means a sequence of one period in the F10 category: alcohol use disorder and 19 periods in the category F43: reaction to severe stress, and adjustment disorders.

**First-time main psychiatric diagnosis - Substance use disorder (SUD) F10 (F10-F19):**

Sequence "Freq" "Percent"

"10/10" 139 45.5737704918033

"60/10" 8 2.62295081967213

"20/10" 6 1.9672131147541

**First-time main psychiatric diagnosis - Schizophrenia spectrum disorder F20 (F20-29)**

Sequence "Freq" "Percent"

"20/10" 320 39.1676866585067

"60/10" 23 2.81517747858017

"43/10" 19 2.32558139534884

"99/10" 11 1.34638922888617

"32/10" 10 1.22399020807834

"40/10" 9 1.1015911872705

"20/8-60/2" 8 0.979192166462668

"84/10" 8 0.979192166462668

"30/10" 7 0.856793145654835

"90/10" 7 0.856793145654835

"20/2-60/8" 6 0.734394124847001

"20/1-43/9" 5 0.611995104039168

"20/1-60/9" 5 0.611995104039168

"20/3-60/7" 5 0.611995104039168

**First-time main psychiatric diagnosis - Bipolar disorder F30 (F30-31)**

Sequence "Freq" "Percent"

"30/10" 29 42.0289855072464

**First-time main psychiatric diagnosis - Single and recurrent depression F32 (F32+F33)**

Sequence "Freq" "Percent"

"32/10" 1252 40.9685863874346

"43/10" 71 2.32329842931937

"60/10" 59 1.93062827225131

"20/10" 57 1.8651832460733

"32/1-43/9" 41 1.34162303664921

"32/8-60/2" 26 0.850785340314136

"32/3-60/7" 25 0.818062827225131

"32/2-60/8" 24 0.785340314136126

"90/10" 24 0.785340314136126

"32/1-60/9" 23 0.75261780104712

"40/10" 23 0.75261780104712

"50/10" 20 0.654450261780105

"84/10" 20 0.654450261780105

"32/5-60/5" 19 0.621727748691099

"32/1-20/9" 18 0.589005235602094

"32/7-60/3" 17 0.556282722513089

"32/3-20/7" 16 0.523560209424084

"32/4-60/6" 15 0.490837696335079

"32/9-60/1" 15 0.490837696335079

"32/2-43/8" 12 0.392670157068063

"32/4-50/6" 12 0.392670157068063

"32/7-43/3" 12 0.392670157068063

"32/1-90/9" 11 0.359947643979058

"32/3-43/7" 11 0.359947643979058

"32/5-20/5" 11 0.359947643979058

"32/5-43/5" 11 0.359947643979058

"32/6-20/4" 11 0.359947643979058

"32/6-43/4" 11 0.359947643979058

"32/7-40/3" 11 0.359947643979058

"32/9-43/1" 11 0.359947643979058

"32/2-40/8" 10 0.327225130890052

"32/3-40/7" 10 0.327225130890052

"32/4-20/6" 10 0.327225130890052

"32/7-20/3" 10 0.327225130890052

"99/10" 10 0.327225130890052

"32/2-20/8" 9 0.294502617801047

"32/5-40/5" 9 0.294502617801047

"32/8-30/2" 9 0.294502617801047

"32/9-40/1" 9 0.294502617801047

"30/10" 8 0.261780104712042

"32/1-40/9" 8 0.261780104712042

"32/1-50/9" 8 0.261780104712042

"32/4-40/6" 8 0.261780104712042

"32/4-43/6" 8 0.261780104712042

"32/6-60/4" 8 0.261780104712042

"32/2-99/8" 7 0.229057591623037

"32/4-90/6" 7 0.229057591623037

"32/3-50/7" 6 0.196335078534031

"32/6-40/4" 6 0.196335078534031

"32/8-40/2" 6 0.196335078534031

"80/10" 6 0.196335078534031

"32/1-84/9" 5 0.163612565445026

"32/2-90/8" 5 0.163612565445026

"32/3-90/7" 5 0.163612565445026

"32/5-50/5" 5 0.163612565445026

"32/9-30/1" 5 0.163612565445026

"42/10" 5 0.163612565445026

**First-time main psychiatric diagnosis - Other mood disorders F34 (F34-F39)**

Sequence "Freq" "Percent"

"34/10" 48 30

"32/10" 9 5.625

"43/10" 8 5

"60/10" 8 5

"40/10" 6 3.75

"20/10" 5 3.125

**First-time main psychiatric diagnosis - Anxiety disorders F40 (F40+F41+F93)**

Sequence "Freq" "Percent"

"40/10" 1260 55.9254327563249

"60/10" 19 0.843320017754106

"40/2-43/8" 16 0.710164225477142

"84/10" 16 0.710164225477142

"99/10" 16 0.710164225477142

"90/10" 15 0.66577896138482

"40/7-60/3" 14 0.621393697292499

"40/8-60/2" 14 0.621393697292499

"43/10" 14 0.621393697292499

"40/5-60/5" 13 0.577008433200177

"94/10" 13 0.577008433200177

"20/10" 12 0.532623169107856

"40/1-43/9" 12 0.532623169107856

"32/10" 10 0.443852640923214

"40/9-32/1" 10 0.443852640923214

"40/2-60/8" 9 0.399467376830892

"40/3-60/7" 9 0.399467376830892

"40/4-43/6" 9 0.399467376830892

"40/9-60/1" 9 0.399467376830892

"40/1-32/9" 8 0.355082112738571

"40/3-43/7" 8 0.355082112738571

"40/4-42/6" 8 0.355082112738571

"40/6-43/4" 8 0.355082112738571

"40/6-60/4" 8 0.355082112738571

"42/10" 8 0.355082112738571

"40/2-20/8" 7 0.310696848646249

"40/2-90/8" 7 0.310696848646249

"40/4-32/6" 7 0.310696848646249

"40/4-60/6" 7 0.310696848646249

"40/7-50/3" 7 0.310696848646249

"40/8-20/2" 7 0.310696848646249

"40/8-32/2" 7 0.310696848646249

"40/9-43/1" 7 0.310696848646249

"40/1-42/9" 6 0.266311584553928

"40/3-20/7" 6 0.266311584553928

"40/3-32/7" 6 0.266311584553928

"40/3-42/7" 6 0.266311584553928

"40/6-20/4" 6 0.266311584553928

"40/1-60/9" 5 0.221926320461607

"40/4-20/6" 5 0.221926320461607

"40/4-84/6" 5 0.221926320461607

"40/5-20/5" 5 0.221926320461607

"40/5-42/5" 5 0.221926320461607

"40/5-50/5" 5 0.221926320461607

"40/5-84/5" 5 0.221926320461607

"40/6-32/4" 5 0.221926320461607

"40/6-42/4" 5 0.221926320461607

"40/6-50/4" 5 0.221926320461607

"40/7-32/3" 5 0.221926320461607

"40/7-90/3" 5 0.221926320461607

"40/8-43/2" 5 0.221926320461607

"50/10" 5 0.221926320461607

**First-time main psychiatric diagnosis - Obsessive-compulsive disorder (OCD) F42 (F42):**

Sequence "Freq" "Percent"

"42/10" 831 61.9686800894855

"40/10" 13 0.969425801640567

"84/10" 10 0.745712155108128

"42/6-50/4" 8 0.596569724086503

"43/10" 8 0.596569724086503

"90/10" 8 0.596569724086503

"20/10" 7 0.52199850857569

"42/7-50/3" 7 0.52199850857569

"42/3-40/7" 6 0.447427293064877

"42/5-32/5" 6 0.447427293064877

"42/5-50/5" 6 0.447427293064877

"60/10" 6 0.447427293064877

"42/1-84/9" 5 0.372856077554064

"42/2-40/8" 5 0.372856077554064

"42/3-50/7" 5 0.372856077554064

"42/4-50/6" 5 0.372856077554064

"42/5-40/5" 5 0.372856077554064

"42/5-60/5" 5 0.372856077554064

"42/7-20/3" 5 0.372856077554064

"42/9-32/1" 5 0.372856077554064

**First-time main psychiatric diagnosis - Stress and adjustment disorders F43 (F43):**

Sequence "Freq" "Percent"

"43/10" 4156 55.0974413363383

"60/10" 90 1.19315922046931

"32/10" 62 0.821954129656635

"43/5-60/5" 61 0.808696804984754

"99/10" 56 0.742410181625348

"43/2-60/8" 55 0.729152856953467

"43/4-60/6" 55 0.729152856953467

"43/1-60/9" 53 0.702638207609704

"43/7-60/3" 51 0.676123558265942

"43/3-60/7" 50 0.662866233594061

"43/6-60/4" 48 0.636351584250298

"20/10" 44 0.583322285562773

"43/9-60/1" 43 0.570064960890892

"43/2-32/8" 41 0.54355031154713

"43/9-32/1" 41 0.54355031154713

"43/5-32/5" 39 0.517035662203367

"43/1-32/9" 38 0.503778337531486

"43/8-60/2" 36 0.477263688187724

"90/10" 34 0.450749038843961

"43/4-32/6" 33 0.43749171417208

"40/10" 31 0.410977064828318

"43/3-32/7" 30 0.397719740156436

"43/6-32/4" 30 0.397719740156436

"43/1-90/9" 29 0.384462415484555

"43/7-32/3" 29 0.384462415484555

"94/10" 29 0.384462415484555

"43/8-32/2" 26 0.344690441468912

"43/3-20/7" 25 0.33143311679703

"43/2-90/8" 23 0.304918467453268

"43/9-40/1" 23 0.304918467453268

"43/2-40/8" 22 0.291661142781387

"43/3-90/7" 21 0.278403818109506

"43/1-20/9" 19 0.251889168765743

"43/5-40/5" 19 0.251889168765743

"43/1-99/9" 18 0.238631844093862

"43/1-40/9" 17 0.225374519421981

"50/10" 17 0.225374519421981

"43/2-20/8" 16 0.212117194750099

"43/4-20/6" 16 0.212117194750099

"43/4-40/6" 16 0.212117194750099

"43/9-90/1" 16 0.212117194750099

"43/7-40/3" 15 0.198859870078218

"43/7-90/3" 15 0.198859870078218

"43/2-50/8" 14 0.185602545406337

"84/10" 14 0.185602545406337

"43/5-0/5" 13 0.172345220734456

"43/3-40/7" 12 0.159087896062575

"43/3-50/7" 12 0.159087896062575

"43/6-0/4" 12 0.159087896062575

"43/8-40/2" 12 0.159087896062575

"0/10" 11 0.145830571390693

"43/2-10/8" 11 0.145830571390693

"43/4-50/6" 11 0.145830571390693

"43/6-40/4" 11 0.145830571390693

"43/8-90/2" 11 0.145830571390693

"43/9-99/1" 11 0.145830571390693

"43/1-50/9" 10 0.132573246718812

"43/6-90/4" 10 0.132573246718812

"43/9-50/1" 10 0.132573246718812

"10/10" 9 0.119315922046931

"42/10" 9 0.119315922046931

"43/2-99/8" 9 0.119315922046931

"43/3-10/7" 9 0.119315922046931

"43/4-10/6" 9 0.119315922046931

"43/4-90/6" 9 0.119315922046931

"43/5-20/5" 9 0.119315922046931

"43/6-20/4" 9 0.119315922046931

"43/9-20/1" 9 0.119315922046931

"43/3-84/7" 8 0.10605859737505

"43/7-20/3" 8 0.10605859737505

"43/8-30/2" 8 0.10605859737505

"43/8-50/2" 8 0.10605859737505

"70/10" 8 0.10605859737505

"80/10" 8 0.10605859737505

"43/1-10/9" 7 0.0928012727031685

"43/2-70/8" 7 0.0928012727031685

"43/4-99/6" 7 0.0928012727031685

"43/5-42/5" 7 0.0928012727031685

"43/5-90/5" 7 0.0928012727031685

"43/5-99/5" 7 0.0928012727031685

"43/6-99/4" 7 0.0928012727031685

"43/3-99/7" 6 0.0795439480312873

"43/5-50/5" 6 0.0795439480312873

"43/8-99/2" 6 0.0795439480312873

"43/9-0/1" 6 0.0795439480312873

"43/9-30/1" 6 0.0795439480312873

"30/10" 5 0.0662866233594061

"32/1-60/9" 5 0.0662866233594061

"43/2-0/8" 5 0.0662866233594061

"43/3-34/7" 5 0.0662866233594061

"43/4-84/6" 5 0.0662866233594061

"43/6-50/4" 5 0.0662866233594061

"43/7-0/3" 5 0.0662866233594061

"43/7-32/1-60/2" 5 0.0662866233594061

"43/7-50/3" 5 0.0662866233594061

"43/8-10/2" 5 0.0662866233594061

"99/3-60/7" 5 0.0662866233594061

**First-time main psychiatric diagnosis - Somatoform disorders F44 (F44-F48):**

Sequence "Freq" "Percent"

"44/10" 154 52.3809523809524

"40/10" 9 3.06122448979592

"43/10" 5 1.70068027210884

**First-time main psychiatric diagnosis - Eating disorders F50 (F50):**

Sequence "Freq" "Percent"

"50/10" 2662 68.2564102564103

"43/10" 31 0.794871794871795

"60/10" 22 0.564102564102564

"50/1-43/9" 18 0.461538461538462

"50/4-60/6" 18 0.461538461538462

"50/3-32/7" 17 0.435897435897436

"32/10" 16 0.41025641025641

"50/3-60/7" 15 0.384615384615385

"50/5-60/5" 15 0.384615384615385

"50/8-60/2" 15 0.384615384615385

"50/2-43/8" 14 0.358974358974359

"50/6-60/4" 14 0.358974358974359

"50/7-60/3" 14 0.358974358974359

"50/9-40/1" 14 0.358974358974359

"50/9-60/1" 14 0.358974358974359

"50/3-43/7" 13 0.333333333333333

"50/8-32/2" 13 0.333333333333333

"50/1-60/9" 12 0.307692307692308

"50/2-32/8" 12 0.307692307692308

"50/4-43/6" 12 0.307692307692308

"50/5-32/5" 12 0.307692307692308

"50/9-32/1" 12 0.307692307692308

"20/10" 11 0.282051282051282

"50/2-60/8" 11 0.282051282051282

"50/5-43/5" 11 0.282051282051282

"50/7-0/3" 11 0.282051282051282

"99/10" 11 0.282051282051282

"50/1-40/9" 10 0.256410256410256

"50/5-20/5" 9 0.230769230769231

"50/6-32/4" 9 0.230769230769231

"50/1-32/9" 8 0.205128205128205

"50/4-20/6" 8 0.205128205128205

"50/4-32/6" 8 0.205128205128205

"50/5-40/5" 8 0.205128205128205

"84/10" 8 0.205128205128205

"50/6-0/4" 7 0.179487179487179

"50/7-32/3" 7 0.179487179487179

"50/7-42/3" 7 0.179487179487179

"50/1-90/9" 6 0.153846153846154

"50/3-20/7" 6 0.153846153846154

"50/7-43/3" 6 0.153846153846154

"50/8-0/2" 6 0.153846153846154

"50/9-0/1" 6 0.153846153846154

"90/10" 6 0.153846153846154

"40/10" 5 0.128205128205128

"50/1-20/9" 5 0.128205128205128

"50/2-90/8" 5 0.128205128205128

"50/3-90/7" 5 0.128205128205128

"50/4-40/6" 5 0.128205128205128

"50/8-40/2" 5 0.128205128205128

"50/8-42/2" 5 0.128205128205128

"50/9-42/1" 5 0.128205128205128

"50/9-43/1" 5 0.128205128205128

**First-time main psychiatric diagnosis - Personality disorders F60 (F60-69):**

Sequence "Freq" "Percent"

"60/10" 706 50.3925767309065

"43/10" 22 1.57030692362598

"20/10" 16 1.14204139900071

"99/10" 15 1.07066381156317

"60/1-43/9" 12 0.856531049250535

"60/2-20/8" 10 0.713775874375446

"60/4-20/6" 10 0.713775874375446

"60/3-43/7" 9 0.642398286937901

"60/9-32/1" 9 0.642398286937901

"60/1-20/9" 8 0.571020699500357

"60/2-43/8" 8 0.571020699500357

"60/7-90/3" 8 0.571020699500357

"32/10" 7 0.499643112062812

"50/10" 7 0.499643112062812

"90/10" 7 0.499643112062812

"60/4-43/6" 6 0.428265524625268

"60/9-43/1" 6 0.428265524625268

"60/3-20/7" 5 0.356887937187723

"60/8-20/2" 5 0.356887937187723

"60/8-32/2" 5 0.356887937187723

"60/8-43/2" 5 0.356887937187723

**First-time main psychiatric diagnosis - Intellectual disability F70 (F70-79):**

Sequence "Freq" "Percent"

"70/10" 574 63.9910813823857

"84/10" 13 1.44927536231884

"70/4-90/6" 8 0.891861761426979

"70/2-90/8" 7 0.780379041248606

"70/1-84/9" 6 0.668896321070234

"70/2-84/8" 6 0.668896321070234

"70/3-90/7" 6 0.668896321070234

"70/5-90/5" 6 0.668896321070234

"70/9-40/1" 6 0.668896321070234

"70/1-90/9" 5 0.557413600891862

"70/3-84/7" 5 0.557413600891862

"70/6-84/4" 5 0.557413600891862

"90/10" 5 0.557413600891862

**First-time main psychiatric diagnosis - Other developmental disorders F80 (F80-83):**

Sequence "Freq" "Percent"

"80/10" 507 59.7877358490566

"70/10" 10 1.17924528301887

"80/3-90/7" 10 1.17924528301887

"90/10" 10 1.17924528301887

"80/2-90/8" 9 1.06132075471698

"84/10" 9 1.06132075471698

"80/5-90/5" 7 0.825471698113208

"80/1-90/9" 6 0.707547169811321

"80/3-43/7" 6 0.707547169811321

"80/6-90/4" 6 0.707547169811321

"80/9-84/1" 6 0.707547169811321

"94/10" 6 0.707547169811321

"80/1-84/9" 5 0.589622641509434

"80/3-84/7" 5 0.589622641509434

"80/7-32/3" 5 0.589622641509434

**First-time main psychiatric diagnosis - Autism spectrum disorder (ASD) F84 (minus F84.2-F84.4):**

Sequence "Freq" "Percent"

"84/10" 1315 66.1136249371543

"90/10" 31 1.55857214680744

"70/10" 29 1.45801910507793

"94/10" 17 0.854700854700855

"84/1-90/9" 15 0.754147812971342

"80/10" 13 0.65359477124183

"84/2-90/8" 11 0.553041729512318

"99/10" 11 0.553041729512318

"84/5-90/5" 10 0.502765208647562

"84/6-90/4" 10 0.502765208647562

"40/10" 9 0.452488687782805

"84/9-90/1" 9 0.452488687782805

"84/1-0/9" 8 0.402212166918049

"84/3-90/7" 8 0.402212166918049

"84/4-90/6" 8 0.402212166918049

"84/7-90/3" 8 0.402212166918049

"84/3-70/7" 7 0.351935646053293

"84/9-43/1" 7 0.351935646053293

"84/7-43/3" 6 0.301659125188537

"0/10" 5 0.251382604323781

"20/10" 5 0.251382604323781

"43/10" 5 0.251382604323781

"84/1-80/9" 5 0.251382604323781

"84/6-43/4" 5 0.251382604323781

"84/8-40/2" 5 0.251382604323781

"84/9-40/1" 5 0.251382604323781

**First-time main psychiatric diagnosis – ADHD F90 (F90 + F98.8):**

Sequence "Freq" "Percent"

"90/10" 2034 64.326375711575

"84/10" 34 1.0752688172043

"90/6-60/4" 19 0.600885515496521

"90/9-60/1" 19 0.600885515496521

"90/4-60/6" 18 0.569259962049336

"90/9-43/1" 17 0.537634408602151

"80/10" 16 0.506008855154965

"90/1-84/9" 16 0.506008855154965

"94/10" 16 0.506008855154965

"90/7-43/3" 14 0.442757748260595

"90/8-60/2" 14 0.442757748260595

"90/6-84/4" 13 0.411132194813409

"90/7-60/3" 13 0.411132194813409

"99/10" 13 0.411132194813409

"90/3-84/7" 12 0.379506641366224

"90/7-84/3" 12 0.379506641366224

"90/8-43/2" 12 0.379506641366224

"90/6-43/4" 11 0.347881087919039

"70/10" 10 0.316255534471853

"90/1-99/9" 10 0.316255534471853

"90/2-84/8" 10 0.316255534471853

"90/4-84/6" 10 0.316255534471853

"40/10" 9 0.284629981024668

"90/1-70/9" 9 0.284629981024668

"90/5-84/5" 9 0.284629981024668

"90/9-32/1" 9 0.284629981024668

"90/2-60/8" 8 0.253004427577483

"90/3-99/7" 8 0.253004427577483

"0/10" 7 0.221378874130297

"43/10" 7 0.221378874130297

"90/4-43/6" 7 0.221378874130297

"90/5-60/5" 7 0.221378874130297

"90/7-40/3" 7 0.221378874130297

"60/10" 6 0.189753320683112

"90/1-60/9" 6 0.189753320683112

"90/2-32/8" 6 0.189753320683112

"90/4-70/6" 6 0.189753320683112

"90/5-99/5" 6 0.189753320683112

"90/6-70/4" 6 0.189753320683112

"90/9-40/1" 6 0.189753320683112

"90/1-0/9" 5 0.158127767235927

"90/6-99/4" 5 0.158127767235927

"90/7-32/3" 5 0.158127767235927

"90/8-70/2" 5 0.158127767235927

"90/8-84/2" 5 0.158127767235927

"90/9-0/1" 5 0.158127767235927

**First-time main psychiatric diagnosis - Conduct disorders F91 (F91):**

Sequence "Freq" "Percent"

"91/10" 196 42.6086956521739

"90/10" 10 2.17391304347826

"94/10" 10 2.17391304347826

"99/10" 10 2.17391304347826

"43/10" 6 1.30434782608696

"60/10" 6 1.30434782608696

"91/1-60/9" 6 1.30434782608696

"91/2-90/8" 5 1.08695652173913

"91/4-60/6" 5 1.08695652173913

**First-time main psychiatric diagnosis - Attachment disorders F94 (minus F94.0):**

Sequence "Freq" "Percent"

"94/10" 540 57.3857598299681

"99/10" 10 1.06269925611052

"84/10" 9 0.956429330499469

"90/10" 7 0.743889479277364

"94/5-60/5" 7 0.743889479277364

"43/10" 6 0.637619553666312

"94/2-90/8" 6 0.637619553666312

"94/4-84/6" 6 0.637619553666312

"94/7-90/3" 6 0.637619553666312

"94/8-43/2" 6 0.637619553666312

"94/8-60/2" 6 0.637619553666312

"94/9-60/1" 6 0.637619553666312

"94/1-90/9" 5 0.53134962805526

"94/2-99/8" 5 0.53134962805526

"94/4-90/6" 5 0.53134962805526

"94/6-84/4" 5 0.53134962805526

**First-time main psychiatric diagnosis - Tic disorders F95 (F95):**

Sequence "Freq" "Percent"

"95/10" 78 49.3670886075949

**First-time main psychiatric diagnosis – Other F99 (F51-F59, F84.2-F84.4, F88, F89, F92,**

**F98.0-F98.6,F98.9,F99.9):**

Sequence "Freq" "Percent"

"99/10" 1304 41.5418923223957

"84/10" 95 3.02644154189232

"43/10" 84 2.67601146862058

"60/10" 75 2.38929595412552

"90/10" 67 2.1344377190188

"40/10" 42 1.33800573431029

"94/10" 42 1.33800573431029

"20/10" 25 0.796431984708506

"80/10" 25 0.796431984708506

"99/1-84/9" 23 0.732717425931825

"99/2-90/8" 21 0.669002867155145

"70/10" 20 0.637145587766805

"99/5-90/5" 20 0.637145587766805

"50/10" 19 0.605288308378464

"32/10" 18 0.573431028990124

"99/1-60/9" 17 0.541573749601784

"99/2-60/8" 16 0.509716470213444

"99/5-60/5" 14 0.446001911436763

"99/1-90/9" 13 0.414144632048423

"99/7-43/3" 13 0.414144632048423

"99/3-60/7" 12 0.382287352660083

"99/4-60/6" 12 0.382287352660083

"99/4-90/6" 12 0.382287352660083

"99/6-60/4" 12 0.382287352660083

"99/7-60/3" 12 0.382287352660083

"91/10" 11 0.350430073271743

"99/1-43/9" 11 0.350430073271743

"99/2-84/8" 11 0.350430073271743

"99/6-90/4" 11 0.350430073271743

"99/3-90/7" 10 0.318572793883402

"99/6-43/4" 10 0.318572793883402

"99/7-40/3" 10 0.318572793883402

"99/8-60/2" 10 0.318572793883402

"99/3-43/7" 9 0.286715514495062

"99/6-84/4" 9 0.286715514495062

"99/8-43/2" 9 0.286715514495062

"99/8-84/2" 9 0.286715514495062

"99/9-43/1" 9 0.286715514495062

"0/10" 8 0.254858235106722

"42/10" 8 0.254858235106722

"99/1-94/9" 8 0.254858235106722

"99/2-40/8" 8 0.254858235106722

"99/2-43/8" 8 0.254858235106722

"99/5-43/5" 8 0.254858235106722

"99/3-70/7" 7 0.223000955718382

"99/6-40/4" 7 0.223000955718382

"99/8-90/2" 7 0.223000955718382

"99/9-32/1" 7 0.223000955718382

"44/10" 6 0.191143676330041

"99/1-40/9" 6 0.191143676330041

"99/7-32/3" 6 0.191143676330041

"99/8-40/2" 6 0.191143676330041

"99/9-60/1" 6 0.191143676330041

"99/2-20/8" 5 0.159286396941701

"99/2-32/8" 5 0.159286396941701

"99/3-32/7" 5 0.159286396941701

"99/4-20/6" 5 0.159286396941701

"99/4-84/6" 5 0.159286396941701

"99/5-32/5" 5 0.159286396941701

"99/5-40/5" 5 0.159286396941701

"99/9-0/1" 5 0.159286396941701

**Supplementary Table 4b:** Exact numbers and percentages in the sequence analyses for boys

The number before the slash is the diagnostic category and the number after the slash is the number of 6-month periods spend in that category. For example, “10/20” means a sequence of 20 periods in the category F10: schizophrenia and “10/1-43/19” means a sequence of one period in the F10 category: alcohol use disorder and 19 periods in the category F43: reaction to severe stress, and adjustment disorders.

**First-time main psychiatric diagnosis - Substance use disorder (SUD) F10 (F10-F19):**

Sequence "Freq" "Percent"

"10/10" 298 50.5942275042445

"20/10" 19 3.2258064516129

"10/1-20/9" 8 1.35823429541596

"10/1-90/9" 8 1.35823429541596

"10/3-20/7" 8 1.35823429541596

"10/2-90/8" 7 1.18845500848896

"10/2-20/8" 6 1.01867572156197

"90/10" 6 1.01867572156197

**First-time main psychiatric diagnosis - Schizophrenia spectrum disorder F20 (F20-29)**

Sequence "Freq" "Percent"

"20/10" 382 45.748502994012

"84/10" 24 2.87425149700599

"90/10" 17 2.03592814371257

"99/10" 13 1.55688622754491

"60/10" 12 1.43712574850299

"10/10" 9 1.07784431137725

"43/10" 9 1.07784431137725

"20/1-84/9" 7 0.838323353293413

"32/10" 7 0.838323353293413

"40/10" 6 0.718562874251497

"20/4-99/6" 5 0.598802395209581

"30/10" 5 0.598802395209581

**First-time main psychiatric diagnosis - Bipolar disorder F30 (F30-31)**

Sequence "Freq" "Percent"

"30/10" 28 44.4444444444444

**First-time main psychiatric diagnosis - Single and recurrent depression F32 (F32+F33)**

Sequence "Freq" "Percent"

"32/10" 584 43.0044182621502

"43/10" 35 2.57731958762887

"20/10" 34 2.50368188512518

"84/10" 34 2.50368188512518

"60/10" 20 1.47275405007364

"40/10" 18 1.32547864506627

"90/10" 16 1.17820324005891

"32/1-90/9" 14 1.03092783505155

"32/1-43/9" 10 0.736377025036819

"32/2-90/8" 10 0.736377025036819

"99/10" 10 0.736377025036819

"80/10" 9 0.662739322533137

"32/1-20/9" 8 0.589101620029455

"32/1-84/9" 8 0.589101620029455

"32/3-20/7" 8 0.589101620029455

"32/6-20/4" 8 0.589101620029455

"32/1-99/9" 7 0.515463917525773

"32/1-60/9" 5 0.368188512518409

"32/2-60/8" 5 0.368188512518409

"32/2-84/8" 5 0.368188512518409

"32/3-84/7" 5 0.368188512518409

"32/3-90/7" 5 0.368188512518409

"32/5-20/5" 5 0.368188512518409

"32/5-84/5" 5 0.368188512518409

"94/10" 5 0.368188512518409

**First-time main psychiatric diagnosis - Other mood disorders F34 (F34-F39)**

Sequence "Freq" "Percent"

"34/10" 47 43.5185185185185

**First-time main psychiatric diagnosis - Anxiety disorders F40 (F40+F41+F93)**

Sequence "Freq" "Percent"

"40/10" 1334 61.9888475836431

"84/10" 39 1.81226765799256

"99/10" 35 1.62639405204461

"90/10" 30 1.39405204460967

"43/10" 22 1.02230483271375

"40/2-84/8" 18 0.836431226765799

"20/10" 13 0.604089219330855

"60/10" 12 0.557620817843866

"40/1-84/9" 11 0.511152416356877

"40/3-84/7" 11 0.511152416356877

"40/3-90/7" 10 0.464684014869888

"40/4-84/6" 10 0.464684014869888

"80/10" 10 0.464684014869888

"32/10" 9 0.4182156133829

"40/2-99/8" 9 0.4182156133829

"42/10" 9 0.4182156133829

"70/10" 9 0.4182156133829

"94/10" 9 0.4182156133829

"40/1-90/9" 8 0.371747211895911

"40/6-84/4" 8 0.371747211895911

"40/9-84/1" 8 0.371747211895911

"40/4-90/6" 7 0.325278810408922

"40/5-84/5" 7 0.325278810408922

"40/5-90/5" 7 0.325278810408922

"40/7-84/3" 7 0.325278810408922

"40/8-20/2" 7 0.325278810408922

"40/1-80/9" 6 0.278810408921933

"40/1-99/9" 6 0.278810408921933

"40/2-90/8" 6 0.278810408921933

"40/3-20/7" 6 0.278810408921933

"40/3-99/7" 6 0.278810408921933

"40/8-43/2" 6 0.278810408921933

"40/3-42/7" 5 0.232342007434944

"40/4-20/6" 5 0.232342007434944

"40/6-90/4" 5 0.232342007434944

"40/7-43/3" 5 0.232342007434944

"40/7-90/3" 5 0.232342007434944

"40/8-32/2" 5 0.232342007434944

"40/8-42/2" 5 0.232342007434944

"40/8-70/2" 5 0.232342007434944

"40/9-60/1" 5 0.232342007434944

**First-time main psychiatric diagnosis - Obsessive-compulsive disorder (OCD) F42 (F42):**

Sequence "Freq" "Percent"

"42/10" 784 65.4970760233918

"84/10" 29 2.42272347535505

"40/10" 15 1.2531328320802

"90/10" 14 1.16959064327485

"20/10" 11 0.918964076858814

"42/1-90/9" 7 0.584795321637427

"80/10" 7 0.584795321637427

"42/1-40/9" 6 0.50125313283208

"42/1-84/9" 6 0.50125313283208

"42/2-84/8" 6 0.50125313283208

"42/5-60/5" 6 0.50125313283208

"99/10" 6 0.50125313283208

"42/2-40/8" 5 0.417710944026733

"42/4-90/6" 5 0.417710944026733

"43/10" 5 0.417710944026733

**First-time main psychiatric diagnosis - Stress and adjustment disorders F43 (F43):**

Sequence "Freq" "Percent"

"43/10" 2337 62.3366230994932

"90/10" 50 1.33368898372899

"99/10" 31 0.826887169911977

"20/10" 30 0.800213390237397

"32/10" 23 0.613496932515337

"60/10" 23 0.613496932515337

"40/10" 22 0.586823152840757

"43/3-90/7" 20 0.533475593491598

"84/10" 19 0.506801813817018

"43/6-90/4" 18 0.480128034142438

"43/4-84/6" 16 0.426780474793278

"43/4-90/6" 16 0.426780474793278

"94/10" 16 0.426780474793278

"43/1-90/9" 15 0.400106695118698

"43/2-84/8" 15 0.400106695118698

"43/2-90/8" 14 0.373432915444118

"43/1-99/9" 13 0.346759135769539

"43/8-32/2" 13 0.346759135769539

"43/5-84/5" 12 0.320085356094959

"43/6-84/4" 12 0.320085356094959

"43/3-84/7" 11 0.293411576420379

"43/5-20/5" 11 0.293411576420379

"43/5-32/5" 11 0.293411576420379

"43/5-60/5" 11 0.293411576420379

"43/5-90/5" 11 0.293411576420379

"43/7-90/3" 11 0.293411576420379

"43/9-60/1" 11 0.293411576420379

"10/10" 10 0.266737796745799

"43/1-10/9" 10 0.266737796745799

"43/1-20/9" 10 0.266737796745799

"43/2-60/8" 10 0.266737796745799

"43/4-20/6" 10 0.266737796745799

"43/7-32/3" 10 0.266737796745799

"43/8-90/2" 10 0.266737796745799

"43/5-99/5" 9 0.240064017071219

"43/1-84/9" 8 0.213390237396639

"43/4-32/6" 8 0.213390237396639

"43/5-40/5" 8 0.213390237396639

"43/6-10/4" 8 0.213390237396639

"43/7-20/3" 8 0.213390237396639

"1/10" 7 0.186716457722059

"43/1-60/9" 7 0.186716457722059

"43/1-94/9" 7 0.186716457722059

"43/3-60/7" 7 0.186716457722059

"43/4-40/6" 7 0.186716457722059

"43/4-60/6" 7 0.186716457722059

"43/6-32/4" 7 0.186716457722059

"43/9-10/1" 7 0.186716457722059

"80/10" 7 0.186716457722059

"43/2-20/8" 6 0.160042678047479

"43/3-20/7" 6 0.160042678047479

"43/4-10/6" 6 0.160042678047479

"43/8-20/2" 6 0.160042678047479

"43/8-40/2" 6 0.160042678047479

"43/8-60/2" 6 0.160042678047479

"43/9-0/1" 6 0.160042678047479

"43/9-40/1" 6 0.160042678047479

"43/9-90/1" 6 0.160042678047479

"42/10" 5 0.133368898372899

"43/1-1/9" 5 0.133368898372899

"43/1-40/9" 5 0.133368898372899

"43/2-40/8" 5 0.133368898372899

"43/2-99/8" 5 0.133368898372899

"43/3-40/7" 5 0.133368898372899

"43/6-20/4" 5 0.133368898372899

"43/6-99/4" 5 0.133368898372899

"43/7-84/3" 5 0.133368898372899

"43/7-99/3" 5 0.133368898372899

"43/9-20/1" 5 0.133368898372899

"43/9-32/1" 5 0.133368898372899

"43/9-84/1" 5 0.133368898372899

**First-time main psychiatric diagnosis - Somatoform disorders F44 (F44-F48):**

Sequence "Freq" "Percent"

"44/10" 85 58.6206896551724

"43/10" 6 4.13793103448276

**First-time main psychiatric diagnosis - Eating disorders F50 (F50):**

Sequence "Freq" "Percent"

"50/10" 237 72.0364741641337

"84/10" 6 1.82370820668693

**First-time main psychiatric diagnosis - Personality disorders F60 (F60-69):**

Sequence "Freq" "Percent"

"60/10" 272 53.125

"90/10" 10 1.953125

"20/10" 6 1.171875

"60/3-20/7" 6 1.171875

"84/10" 6 1.171875

"99/10" 6 1.171875

"40/10" 5 0.9765625

"43/10" 5 0.9765625

**First-time main psychiatric diagnosis - Intellectual disability F70 (F70-79):**

Sequence "Freq" "Percent"

"70/10" 1238 65.1236191478169

"84/10" 32 1.68332456601789

"70/2-90/8" 25 1.31509731720147

"90/10" 25 1.31509731720147

"70/3-90/7" 21 1.10468174644924

"70/5-90/5" 20 1.05207785376118

"70/2-84/8" 19 0.999473961073119

"70/3-84/7" 18 0.94687006838506

"70/1-84/9" 17 0.894266175697002

"70/1-90/9" 16 0.841662283008943

"70/6-84/4" 16 0.841662283008943

"70/5-84/5" 14 0.736454497632825

"70/8-90/2" 14 0.736454497632825

"70/4-90/6" 13 0.683850604944766

"70/8-84/2" 12 0.631246712256707

"70/6-90/4" 11 0.578642819568648

"70/4-84/6" 10 0.526038926880589

"70/7-90/3" 10 0.526038926880589

"70/7-84/3" 9 0.47343503419253

"80/10" 8 0.420831141504471

"70/5-43/5" 6 0.315623356128353

"70/7-99/3" 6 0.315623356128353

"99/10" 6 0.315623356128353

"0/10" 5 0.263019463440295

"70/1-94/9" 5 0.263019463440295

"70/2-0/8" 5 0.263019463440295

"70/3-80/7" 5 0.263019463440295

"70/6-40/4" 5 0.263019463440295

"70/9-84/1" 5 0.263019463440295

"70/9-90/1" 5 0.263019463440295

**First-time main psychiatric diagnosis - Other developmental disorders F80 (F80-83):**

Sequence "Freq" "Percent"

"80/10" 1706 61.6106897797039

"90/10" 51 1.84182015167931

"84/10" 42 1.51679306608884

"80/1-90/9" 36 1.30010834236186

"80/5-90/5" 36 1.30010834236186

"80/2-90/8" 35 1.2639942217407

"80/4-90/6" 31 1.11953773925605

"80/3-90/7" 30 1.08342361863489

"80/2-84/8" 28 1.01119537739256

"80/7-90/3" 27 0.975081256771398

"99/10" 25 0.902853015529072

"80/4-84/6" 23 0.830624774286746

"80/6-90/4" 21 0.75839653304442

"80/3-84/7" 20 0.722282412423258

"70/10" 19 0.686168291802095

"80/7-84/3" 19 0.686168291802095

"80/5-84/5" 18 0.650054171180932

"80/6-84/4" 18 0.650054171180932

"80/9-84/1" 16 0.577825929938606

"80/1-84/9" 13 0.469483568075117

"80/9-90/1" 12 0.433369447453954

"80/2-70/8" 11 0.397255326832792

"80/8-84/2" 11 0.397255326832792

"80/8-90/2" 11 0.397255326832792

"94/10" 10 0.361141206211629

"40/10" 9 0.325027085590466

"43/10" 8 0.288912964969303

"80/1-70/9" 8 0.288912964969303

"80/4-70/6" 8 0.288912964969303

"80/7-70/3" 8 0.288912964969303

"80/5-99/5" 7 0.25279884434814

"80/6-43/4" 7 0.25279884434814

"80/2-0/8" 6 0.216684723726977

"80/3-70/7" 6 0.216684723726977

"80/8-43/2" 6 0.216684723726977

"0/10" 5 0.180570603105814

"80/2-43/8" 5 0.180570603105814

"80/4-99/6" 5 0.180570603105814

"80/5-43/5" 5 0.180570603105814

"80/5-70/5" 5 0.180570603105814

"80/9-32/1" 5 0.180570603105814

"91/10" 5 0.180570603105814

**First-time main psychiatric diagnosis - Autism spectrum disorder (ASD) F84 (minus F84.2-F84.4):**

Sequence "Freq" "Percent"

"84/10" 6460 74.8985507246377

"90/10" 145 1.68115942028986

"80/10" 82 0.950724637681159

"70/10" 69 0.8

"84/1-90/9" 64 0.742028985507246

"84/6-90/4" 56 0.649275362318841

"84/3-90/7" 54 0.626086956521739

"84/2-90/8" 52 0.602898550724638

"84/5-90/5" 50 0.579710144927536

"99/10" 49 0.568115942028986

"94/10" 45 0.521739130434783

"84/7-90/3" 43 0.498550724637681

"84/4-90/6" 41 0.47536231884058

"84/8-90/2" 40 0.463768115942029

"84/9-90/1" 28 0.32463768115942

"40/10" 26 0.301449275362319

"0/10" 19 0.220289855072464

"84/1-0/9" 18 0.208695652173913

"84/1-80/9" 18 0.208695652173913

"43/10" 17 0.197101449275362

"84/1-70/9" 15 0.173913043478261

"84/3-0/7" 15 0.173913043478261

"84/9-43/1" 15 0.173913043478261

"84/6-70/4" 12 0.139130434782609

"84/7-32/3" 12 0.139130434782609

"84/8-70/2" 12 0.139130434782609

"84/9-20/1" 12 0.139130434782609

"20/10" 11 0.127536231884058

"84/6-32/4" 11 0.127536231884058

"84/8-20/2" 11 0.127536231884058

"84/8-43/2" 11 0.127536231884058

"84/9-70/1" 11 0.127536231884058

"84/4-20/6" 10 0.115942028985507

"84/6-20/4" 10 0.115942028985507

"84/8-32/2" 10 0.115942028985507

"84/9-32/1" 10 0.115942028985507

"84/1-99/9" 9 0.104347826086957

"84/5-20/5" 9 0.104347826086957

"84/5-43/5" 9 0.104347826086957

"84/6-0/4" 9 0.104347826086957

"84/7-0/3" 9 0.104347826086957

"84/7-43/3" 9 0.104347826086957

"91/10" 9 0.104347826086957

"84/1-40/9" 8 0.0927536231884058

"84/2-70/8" 8 0.0927536231884058

"84/4-70/6" 8 0.0927536231884058

"84/4-99/6" 8 0.0927536231884058

"84/7-20/3" 8 0.0927536231884058

"84/1-20/9" 7 0.0811594202898551

"84/1-94/9" 7 0.0811594202898551

"84/2-0/8" 7 0.0811594202898551

"84/2-20/8" 7 0.0811594202898551

"84/3-20/7" 7 0.0811594202898551

"84/4-0/6" 7 0.0811594202898551

"84/4-32/6" 7 0.0811594202898551

"84/5-70/5" 7 0.0811594202898551

"84/6-40/4" 7 0.0811594202898551

"84/6-42/4" 7 0.0811594202898551

"84/9-42/1" 7 0.0811594202898551

"90/2-84/8" 7 0.0811594202898551

"60/10" 6 0.0695652173913043

"84/2-99/8" 6 0.0695652173913043

"84/3-42/7" 6 0.0695652173913043

"84/4-40/6" 6 0.0695652173913043

"84/5-90/1-84/4" 6 0.0695652173913043

"84/6-43/4" 6 0.0695652173913043

"84/8-0/2" 6 0.0695652173913043

"84/8-40/2" 6 0.0695652173913043

"84/8-60/2" 6 0.0695652173913043

"84/8-99/2" 6 0.0695652173913043

"84/1-32/9" 5 0.0579710144927536

"84/2-40/8" 5 0.0579710144927536

"84/3-32/7" 5 0.0579710144927536

"84/3-40/7" 5 0.0579710144927536

"84/3-43/7" 5 0.0579710144927536

"84/3-70/7" 5 0.0579710144927536

"84/3-99/7" 5 0.0579710144927536

"84/4-43/6" 5 0.0579710144927536

"84/5-0/5" 5 0.0579710144927536

"84/5-32/5" 5 0.0579710144927536

"84/5-99/5" 5 0.0579710144927536

"84/6-99/4" 5 0.0579710144927536

"84/9-60/1" 5 0.0579710144927536

**First-time main psychiatric diagnosis – ADHD F90 (F90 + F98.8):**

Sequence "Freq" "Percent"

"90/10" 8262 74.8980146858852

"84/10" 187 1.6952225546188

"80/10" 69 0.625509926570574

"90/6-84/4" 68 0.616444565315928

"90/1-84/9" 65 0.58924848155199

"99/10" 63 0.571117759042698

"90/3-84/7" 55 0.49859486900553

"90/7-84/3" 55 0.49859486900553

"90/2-84/8" 54 0.489529507750884

"70/10" 47 0.426071978968362

"90/4-84/6" 47 0.426071978968362

"90/5-84/5" 44 0.398875895204424

"90/8-84/2" 41 0.371679811440486

"94/10" 41 0.371679811440486

"91/10" 34 0.308222282657964

"90/9-43/1" 33 0.299156921403318

"90/9-84/1" 32 0.290091560148672

"90/8-43/2" 25 0.22663403136615

"90/6-43/4" 24 0.217568670111504

"90/1-80/9" 22 0.199437947602212

"90/2-99/8" 20 0.18130722509292

"90/7-43/3" 20 0.18130722509292

"0/10" 19 0.172241863838274

"90/1-70/9" 19 0.172241863838274

"90/8-20/2" 19 0.172241863838274

"90/7-10/3" 18 0.163176502583628

"90/8-10/2" 18 0.163176502583628

"43/10" 17 0.154111141328982

"90/2-0/8" 16 0.145045780074336

"90/9-32/1" 16 0.145045780074336

"90/1-99/9" 15 0.13598041881969

"90/7-70/3" 15 0.13598041881969

"90/9-10/1" 15 0.13598041881969

"90/1-94/9" 14 0.126915057565044

"90/4-99/6" 14 0.126915057565044

"90/8-70/2" 14 0.126915057565044

"40/10" 13 0.117849696310398

"90/3-70/7" 13 0.117849696310398

"90/5-43/5" 13 0.117849696310398

"90/7-20/3" 13 0.117849696310398

"90/3-80/7" 12 0.108784335055752

"90/4-20/6" 12 0.108784335055752

"90/9-40/1" 12 0.108784335055752

"95/10" 12 0.108784335055752

"90/6-60/4" 11 0.099718973801106

"90/9-20/1" 11 0.099718973801106

"90/9-70/1" 11 0.099718973801106

"90/1-0/9" 10 0.09065361254646

"90/2-80/8" 10 0.09065361254646

"90/3-43/7" 10 0.09065361254646

"90/3-99/7" 10 0.09065361254646

"90/4-10/6" 10 0.09065361254646

"90/4-80/6" 10 0.09065361254646

"90/5-70/5" 10 0.09065361254646

"90/5-99/5" 10 0.09065361254646

"90/6-20/4" 10 0.09065361254646

"90/8-0/2" 10 0.09065361254646

"90/8-99/2" 10 0.09065361254646

"90/9-60/1" 10 0.09065361254646

"90/2-70/8" 9 0.081588251291814

"90/3-0/7" 9 0.081588251291814

"90/6-32/4" 9 0.081588251291814

"90/6-70/4" 9 0.081588251291814

"90/7-60/3" 9 0.081588251291814

"90/9-99/1" 9 0.081588251291814

"84/2-90/8" 8 0.072522890037168

"84/7-90/3" 8 0.072522890037168

"90/1-91/9" 8 0.072522890037168

"90/5-0/5" 8 0.072522890037168

"90/6-80/4" 8 0.072522890037168

"90/7-32/3" 8 0.072522890037168

"90/9-0/1" 8 0.072522890037168

"20/10" 7 0.063457528782522

"80/4-90/6" 7 0.063457528782522

"90/1-43/9" 7 0.063457528782522

"90/2-43/8" 7 0.063457528782522

"90/2-94/8" 7 0.063457528782522

"90/4-94/6" 7 0.063457528782522

"90/5-20/5" 7 0.063457528782522

"90/5-60/5" 7 0.063457528782522

"90/7-0/3" 7 0.063457528782522

"90/7-40/3" 7 0.063457528782522

"90/7-99/3" 7 0.063457528782522

"90/8-60/2" 7 0.063457528782522

"90/1-10/9" 6 0.054392167527876

"90/3-91/7" 6 0.054392167527876

"90/4-0/6" 6 0.054392167527876

"90/4-43/6" 6 0.054392167527876

"90/4-91/6" 6 0.054392167527876

"90/5-10/5" 6 0.054392167527876

"90/6-99/4" 6 0.054392167527876

"90/8-1/2" 6 0.054392167527876

"90/8-32/2" 6 0.054392167527876

"90/8-40/2" 6 0.054392167527876

"10/10" 5 0.04532680627323

"60/10" 5 0.04532680627323

"80/5-90/5" 5 0.04532680627323

"84/5-90/5" 5 0.04532680627323

"84/6-90/4" 5 0.04532680627323

"90/1-40/9" 5 0.04532680627323

"90/2-20/8" 5 0.04532680627323

"90/2-84/4-90/4" 5 0.04532680627323

"90/2-95/8" 5 0.04532680627323

"90/3-1/7" 5 0.04532680627323

"90/3-10/7" 5 0.04532680627323

"90/3-20/7" 5 0.04532680627323

"90/3-40/7" 5 0.04532680627323

"90/4-70/6" 5 0.04532680627323

"90/5-84/1-90/4" 5 0.04532680627323

"90/6-0/4" 5 0.04532680627323

"90/6-10/4" 5 0.04532680627323

"90/7-42/3" 5 0.04532680627323

"90/7-43/1-90/2" 5 0.04532680627323

"90/9-1/1" 5 0.04532680627323

"90/9-30/1" 5 0.04532680627323

"99/5-90/5" 5 0.04532680627323

**First-time main psychiatric diagnosis - Conduct disorders F91 (F91):**

Sequence "Freq" "Percent"

"91/10" 810 49.5716034271726

"90/10" 52 3.18237454100367

"84/10" 39 2.38678090575275

"94/10" 37 2.26438188494492

"99/10" 32 1.95838433292534

"91/1-90/9" 21 1.28518971848225

"91/2-90/8" 20 1.22399020807834

"91/3-90/7" 20 1.22399020807834

"91/8-90/2" 15 0.917992656058752

"91/9-90/1" 14 0.856793145654835

"43/10" 11 0.673194614443084

"40/10" 10 0.611995104039168

"60/10" 10 0.611995104039168

"91/5-90/5" 10 0.611995104039168

"91/7-43/3" 10 0.611995104039168

"80/10" 9 0.550795593635251

"91/1-99/9" 9 0.550795593635251

"91/4-90/6" 9 0.550795593635251

"70/10" 8 0.489596083231334

"91/1-84/9" 8 0.489596083231334

"91/7-10/3" 8 0.489596083231334

"91/7-90/3" 7 0.428396572827417

"91/3-84/7" 6 0.367197062423501

"91/6-70/4" 6 0.367197062423501

"91/6-84/4" 6 0.367197062423501

"91/6-90/4" 6 0.367197062423501

"91/7-60/3" 6 0.367197062423501

"91/2-84/8" 5 0.305997552019584

"91/5-60/5" 5 0.305997552019584

"91/9-10/1" 5 0.305997552019584

**First-time main psychiatric diagnosis - Attachment disorders F94 (minus F94.0):**

Sequence "Freq" "Percent"

"94/10" 992 60.4141291108404

"84/10" 23 1.40073081607795

"90/10" 23 1.40073081607795

"94/3-90/7" 21 1.278928136419

"94/1-90/9" 18 1.09622411693057

"94/5-90/5" 17 1.0353227771011

"94/4-90/6" 16 0.97442143727162

"94/8-90/2" 16 0.97442143727162

"94/2-90/8" 15 0.913520097442144

"94/4-84/6" 13 0.791717417783191

"94/6-90/4" 13 0.791717417783191

"99/10" 13 0.791717417783191

"94/2-84/8" 12 0.730816077953715

"94/7-90/3" 11 0.669914738124239

"94/7-84/3" 9 0.548112058465286

"94/9-43/1" 9 0.548112058465286

"94/9-90/1" 9 0.548112058465286

"94/1-84/9" 7 0.426309378806334

"94/3-84/7" 7 0.426309378806334

"94/9-84/1" 7 0.426309378806334

"43/10" 6 0.365408038976857

"94/5-84/5" 6 0.365408038976857

"94/6-84/4" 6 0.365408038976857

"94/8-84/2" 6 0.365408038976857

"94/9-10/1" 6 0.365408038976857

"94/9-20/1" 6 0.365408038976857

"70/10" 5 0.304506699147381

"94/5-20/5" 5 0.304506699147381

"94/6-20/4" 5 0.304506699147381

"94/6-60/4" 5 0.304506699147381

"94/6-70/4" 5 0.304506699147381

"94/8-99/2" 5 0.304506699147381

"94/9-32/1" 5 0.304506699147381

**First-time main psychiatric diagnosis - Tic disorders F95 (F95):**

Sequence "Freq" "Percent"

"95/10" 494 61.3664596273292

"90/10" 21 2.60869565217391

"84/10" 19 2.36024844720497

"95/1-90/9" 13 1.61490683229814

"95/3-90/7" 13 1.61490683229814

"95/5-90/5" 12 1.49068322981366

"95/6-90/4" 9 1.11801242236025

"95/2-90/8" 8 0.993788819875776

"95/5-84/5" 8 0.993788819875776

"95/2-40/8" 5 0.62111801242236

"95/4-90/6" 5 0.62111801242236

"95/7-90/3" 5 0.62111801242236

"95/8-90/2" 5 0.62111801242236

**First-time main psychiatric diagnosis – Other F99 (F51-F59, F84.2-F84.4, F88, F89, F92,**

**F98.0-F98.6, F98.9, F99.9):**

Sequence "Freq" "Percent"

"99/10" 2434 46.0374503499149

"84/10" 311 5.88235294117647

"90/10" 251 4.74749385284661

"80/10" 79 1.49423113296766

"94/10" 78 1.47531681482883

"99/1-84/9" 75 1.41857386041233

"99/1-90/9" 64 1.21051636088519

"43/10" 54 1.02137317949688

"40/10" 50 0.945715906941555

"99/2-90/8" 46 0.87005863438623

"99/4-90/6" 44 0.832229998108568

"99/3-90/7" 43 0.813315679969737

"91/10" 37 0.69982977113675

"20/10" 34 0.643086816720257

"60/10" 34 0.643086816720257

"99/3-84/7" 34 0.643086816720257

"99/5-90/5" 34 0.643086816720257

"99/6-84/4" 32 0.605258180442595

"99/6-90/4" 31 0.586343862303764

"70/10" 30 0.567429544164933

"99/2-84/8" 29 0.548515226026102

"99/7-90/3" 26 0.491772271609608

"99/5-84/5" 25 0.472857953470777

"99/8-90/2" 25 0.472857953470777

"99/4-84/6" 24 0.453943635331946

"99/9-90/1" 20 0.378286362776622

"99/1-80/9" 19 0.359372044637791

"99/8-84/2" 18 0.34045772649896

"32/10" 15 0.283714772082466

"99/1-70/9" 15 0.283714772082466

"95/10" 14 0.264800453943635

"99/7-84/3" 14 0.264800453943635

"99/9-84/1" 14 0.264800453943635

"99/8-60/2" 13 0.245886135804804

"99/1-94/9" 11 0.208057499527142

"10/10" 9 0.17022886324948

"99/1-40/9" 9 0.17022886324948

"99/2-70/8" 9 0.17022886324948

"99/9-32/1" 9 0.17022886324948

"99/9-43/1" 9 0.17022886324948

"99/3-0/7" 8 0.151314545110649

"99/5-91/5" 8 0.151314545110649

"99/6-20/4" 8 0.151314545110649

"99/7-10/3" 8 0.151314545110649

"99/7-43/3" 8 0.151314545110649

"99/8-10/2" 8 0.151314545110649

"99/8-43/2" 8 0.151314545110649

"99/9-10/1" 8 0.151314545110649

"99/1-91/9" 7 0.132400226971818

"99/4-20/6" 7 0.132400226971818

"99/5-20/5" 7 0.132400226971818

"99/7-60/3" 7 0.132400226971818

"99/8-20/2" 7 0.132400226971818

"99/9-20/1" 7 0.132400226971818

"80/2-90/8" 6 0.113485908832987

"99/2-40/8" 6 0.113485908832987

"99/3-91/7" 6 0.113485908832987

"99/4-40/6" 6 0.113485908832987

"99/4-80/6" 6 0.113485908832987

"99/6-0/4" 6 0.113485908832987

"99/6-10/4" 6 0.113485908832987

"42/10" 5 0.0945715906941555

"84/2-90/8" 5 0.0945715906941555

"84/4-90/6" 5 0.0945715906941555

"90/5-84/5" 5 0.0945715906941555

"94/7-84/3" 5 0.0945715906941555

"99/1-20/9" 5 0.0945715906941555

"99/1-60/9" 5 0.0945715906941555

"99/2-0/8" 5 0.0945715906941555

"99/2-80/8" 5 0.0945715906941555

"99/3-20/7" 5 0.0945715906941555

"99/3-80/7" 5 0.0945715906941555

"99/4-43/6" 5 0.0945715906941555

"99/4-60/6" 5 0.0945715906941555

"99/4-70/6" 5 0.0945715906941555

"99/4-94/6" 5 0.0945715906941555

"99/5-32/5" 5 0.0945715906941555

"99/7-40/3" 5 0.0945715906941555

"99/8-40/2" 5 0.0945715906941555

**Supplementary Text – Mortality**

All mortality rates were lower than 3%, and the highest mortality rates were seen among girls with intellectual disability (1.6%) and boys with substance use disorder (2.9%). In HR analyses for boys, the highest HRs of death within 10 years of the first diagnosis were identified for D1s of substance abuse (HR=4.92 (1.97; 12.2)), schizophrenia spectrum disorder (HR=3.68 (1.51; 8.95)), and personality disorder (HR=3.57 (1.16; 10.99)). In girls, the highest HRs of death within 10 years of the first diagnosis was identified for D1s of intellectual disability (HR=3.66 (1.25; 10.71)). Concerning mortality, increased rates as found among girls with intellectual disability and boys with substance use disorder in our sample might have different causes, and it should be noted that absolute percentages in ten-year mortality are low. Among children with intellectual disability, previous studies have shown increased mortality rates related to epilepsy and respiratory infections, while for substance use disorder, previous studies have found increased mortality rates to be related to homicide and suicide and motor vehicle accidents. ^1–3^

1 Hughes-McCormack L, Rydzewska E, Cooper S-A, *et al.* 606 Rates, causes and predictors of mortality and avoidable mortality in children and young people with and without intellectual disabilities. *Arch Dis Child* 2023; **108**: A92–3.

2 Border R, Corley RP, Brown SA, *et al.* Independent predictors of mortality in adolescents ascertained for conduct disorder and substance use problems, their siblings and community controls. *Addiction* 2018; **113**: 2107–15.

3 Erskine HE, Moffitt TE, Copeland WE, *et al.* A heavy burden on young minds: the global burden of mental and substance use disorders in children and youth. *Psychol Med* 2015; **45**: 1551–63.
